# DCAF16‐Based Covalent Molecular Glues for Targeted Protein Degradation of Histone Deacetylases

**DOI:** 10.1002/ardp.70045

**Published:** 2025-07-09

**Authors:** Tao Sun, Shiyang Zhai, Stephan Lepper, Beate König, Mateo Malenica, Irina Honin, Finn K. Hansen

**Affiliations:** ^1^ Department of Pharmaceutical and Cell Biological Chemistry, Pharmaceutical Institute University of Bonn Bonn Germany

**Keywords:** cancer, DCAF16, histone deacetylases (HDACs), molecular glues, targeted protein degradation (TPD)

## Abstract

Histone deacetylases (HDACs) are intriguing cancer targets due to their high expression in many tumors. Consequently, inhibition or degradation of HDACs can be beneficial for cancer therapy. Targeted protein degradation using molecular glues represents a promising therapeutic approach, enabling the specific degradation of numerous disease‐causing proteins. However, the rational design of molecular glues in a target‐based manner remains challenging. A recent study has described the identification of a DCAF16‐based covalent linker‐less chemical handle for molecular glues. This covalent warhead can be attached to protein of interest ligands to induce the targeted degradation of various protein classes. Inspired by this, we designed and synthesized a new class of DCAF16‐based covalent molecular glues utilizing different zinc‐binding groups for the targeted degradation of HDACs. This approach led to the discovery of an efficient molecular glue (**10a**) that reduced HDAC1 levels in multiple myeloma MM.1S cells in a potent and preferential manner.

## Graphical abstract

Inspired by the recently identified vinylsulfonyl piperazine handle, which enables conjugation to protein of interest ligands for targeted protein degradation, a novel class of DCAF16‐based covalent molecular glues to degrade histone deacetylases (HDACs) was developed. This strategy yielded an effective molecular glue (**10a**) that significantly and preferentially reduced HDAC1 levels in MM.1S cells.

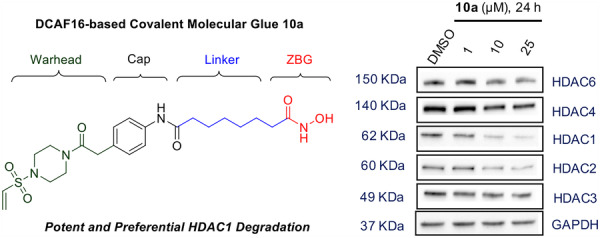

## 1 Introduction

Histone acetylation is regulated by histone deacetylases (HDACs) and histone acetyltransferases (HATs). Modulating HDAC levels has been shown to influence many cellular processes including cell growth, cell cycle, and chromatin decondensation [1]. The HDAC family encompasses 18 isoforms classified into four groups: Class I (HDAC1, 2, 3, and 8), Class IIa (HDAC4, 5, 7, and 9), Class IIb (HDAC6 and 10), Class III (Sirt1–7), and Class IV (HDAC11) [1]. HDACs are intriguing cancer targets due to their overexpression in many tumors. Consequently, inhibition or degradation of HDACs can be beneficial for cancer treatment. To date, four anticancer HDAC inhibitors have been approved by the FDA for the treatment of T‐cell lymphoma and multiple myeloma [2]. Additionally, novel HDAC‐based therapeutic strategies have emerged in recent years, underscoring the broad potential applications of HDAC‐targeted therapies [3, 4, 5].

Targeted protein degradation (TPD) using molecular glues and proteolysis‐targeting chimeras (PROTACs) represents a promising therapeutic strategy, enabling the selective degradation of numerous disease‐causing proteins [6]. Compared to heterobifunctional PROTACs, molecular glue degraders are particularly promising due to their lower molecular weights and favorable drug‐like properties. However, unlike PROTACs, which can be rationally designed, the majority of molecular glue degraders have been identified serendipitously or through phenotypic screening methods. The rational design of molecular glue degraders in a target‐specific context remains a significant challenge, limiting the broader applications of molecular glues [7, 8, 9]. In addition to the complex design of molecular glues, they have several other limitations compared to small‐molecule inhibitors. For instance, not all proteins are degradable because they are poorly recognized by E3 ligases or lack a suitable ubiquitination site. In addition, for proteins with short half‐lives and high turnover rates, the relative advantage of a molecular glue may be less pronounced. However, despite these challenges associated with the development of molecular glues, they also possess advantages over small‐molecule inhibitors: Molecular glues (1) can exploit shallow protein−protein interfaces between E3 ligases and therapeutic proteins that may lack deep binding pockets, which presents a significant advantage in drug development, (2) act via a catalytic mode of action, (3) remove all functions of the target protein, not just enzymatic activity, but also scaffolding or noncatalytic roles, and (4) exhibit sustained effects, particularly for proteins with moderate and long half‐lives [10, 11].

A recent study has described the identification of a DDB1‐ and CUL4‐associated factor 16 (DCAF16)‐based covalent, degradative, and linker‐less chemical handle [12]. This vinylsulfonyl piperazine handle can be conjugated to protein of interest (POI) ligands to induce the degradation of various proteins. Building on the successful applications of this chemical handle, we designed and synthesized a novel class of DCAF16‐based covalent molecular glues utilizing various zinc‐binding groups (ZBGs) for the targeted degradation of HDACs. We then investigated the degradation efficacy and isoform selectivity of the molecular glue degraders. Additionally, the antiproliferative activity, HDAC inhibition, and induction of apoptosis were further evaluated in MM.1S cells.

## 2 Results and Discussion

### 2.1 Chemistry

**Figure 1:**
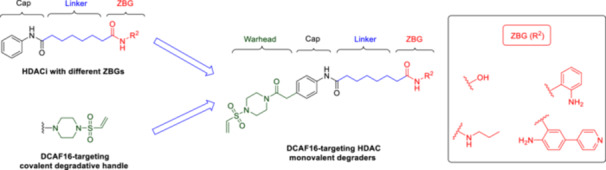
Rational design of DCAF16‐based HDAC molecular glues.

To investigate whether the DCAF16‐based covalent handle can convert nondegrading HDAC inhibitors into degraders, we designed a series of molecular glues for targeting HDACs. Traditionally, HDAC inhibitors, such as vorinostat, consist of three key components: a cap structure, a ZBG, and a linker connecting the cap to the ZBG [1]. Among these components, the cap structure can exhibit considerable structural diversity, allowing for the design of HDAC inhibitors with a wide range of structures [13]. Accordingly, the covalent handle was introduced to the cap group of the HDAC inhibitor vorinostat. Additionally, the hydroxamic acid of vorinostat was substituted with three different ZBGs for further explorations (Figure 1).

**Scheme 1:**
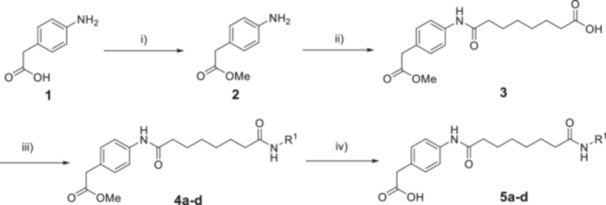
Synthesis of HDAC ligands **5a–d**. Reagents and conditions: (i) SOCl_2_, CH_3_OH, 80°C, 16 h, 97% yield. (ii) Suberic anhydride, NaHCO_3_, THF, rt, 16 h, 63% yield. (iii) R^1^‐NH_2_, HATU, DIPEA, DMF, rt, 16 h, 28%–88% yield. (iv) LiOH·H_2_O, THF/H_2_O (v/v = 1:1), rt, 2–12 h, 26%–84% yield.

**Scheme 2:**
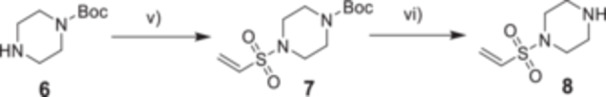
Synthesis of the DCAF16 warhead **8**. Reagents and conditions: (v) 2‐chloroethanesulfonyl chloride, TEA, DCM, 0°C → rt, 16 h. (vi) TFA, DCM, rt, 1 h, 95% yield (over two steps).

**Scheme 3:**
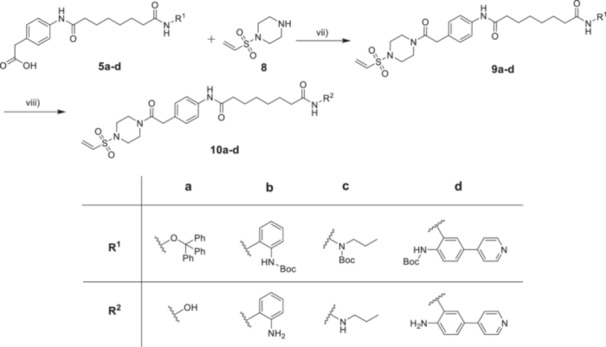
Synthesis of DCAF16‐based HDAC molecular glues **10a–d**. Reagents and conditions: (vii) HATU, DIPEA, DMF, rt, 16 h, 33%–47% yield. (viii) TFA, DCM, rt, 1 h, 25%–85% yield.

The synthetic routes for the target Compounds **10a–d** are outlined in Schemes 1, 2, 3. Briefly, for the synthesis of the HDAC ligands (Scheme 1), 4‐aminophenylacetic acid (**1**) was first activated with thionyl chloride and esterified with methanol to yield intermediate **2**, which was further treated with suberic anhydride to afford **3**. Subsequently, Compound **3** was subjected to amide coupling reactions with various ZBG precursors to produce Compounds **4a–d**. The protected HDAC ligands **5a–d** with a free phenylacetic acid moiety were prepared by the treatment of **4a–d** with LiOH·H_2_O [14].

For the synthesis of the DCAF16 warhead (Scheme 2), Compound **6** was reacted with 2‐chloroethanesulfonyl chloride in the presence of triethylamine (TEA) in dry dichloromethane to give **7**, which was then directly treated with trifluoroacetic acid (TFA) to yield precursor **8** [12].

Next, **5a–d** were conjugated with **8** using HATU and DIPEA as amide coupling system to produce **9a–d**. Finally, deprotection of **9a–d** with TFA afforded the target Compounds **10a–d** (Scheme 3).

### 2.2 Biological Evaluation

#### 2.2.1 HDAC Degradation by DCAF16‐Recruiting Covalent Molecular Glues **10a–d**

**Figure 2:**
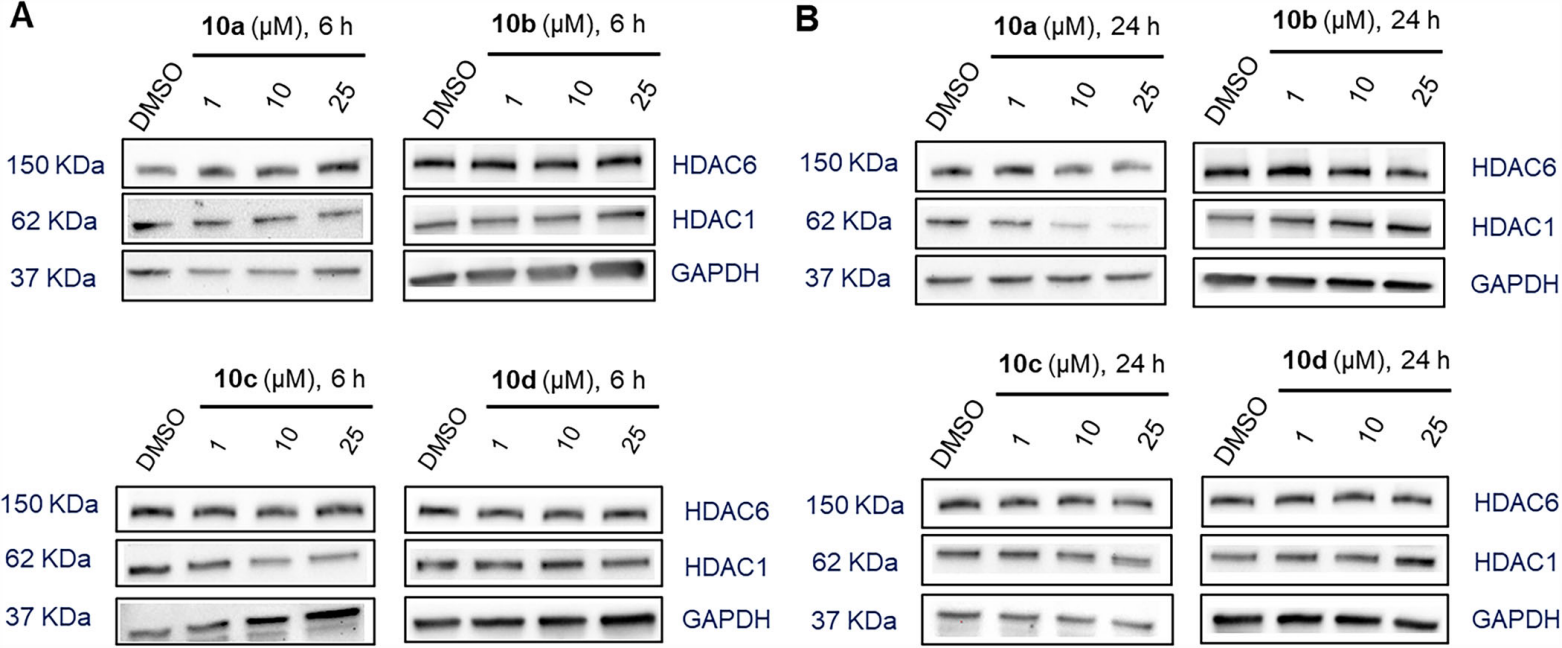
Degradation of HDAC1 and HDAC6 mediated by **10a–d**. MM.1S cells were treated with Compounds **10a–d** at concentrations of 1, 10, and 25 μM for 6 h (A) or for 24 h (B), with DMSO as vehicle control. HDAC1 and HDAC6 levels were detected by immunoblot analysis. GAPDH was used as the loading control. Representative images from a total of *n* = 2 replicates.

First, the multiple myeloma cell line MM.1S was treated with varying concentrations (1, 10, and 25 µM) of degraders **10a–d** for 6 and 24 h, respectively. HDAC1 and HDAC6 were selected for investigation due to their critical roles in various diseases such as cancer [15]. HDAC1 and HDAC6 protein levels were subsequently evaluated using western blot analysis. As summarized in Figure 2A and Supporting Information S2: Figure S1, all compounds exhibited either no or weak degradation efficacy for HDAC1 and HDAC6 at different concentrations after 6 h. This limited efficacy could be due to insufficient incubation time of the compounds with MM.1S cells. To address this, the incubation time was extended to 24 h to investigate whether **10a–d** displayed enhanced degradation of HDAC1 and HDAC6 after longer treatment times (Figure 2B and Supporting Information S2: Figure S2). All compounds still exerted no degradation of HDAC6 at different concentrations after 24 h of treatment. However, different from **10b–d**, degrader **10a**, which contains a hydroxamic acid as ZBG, achieved substantial degradation of HDAC1 with a maximal degradation (*D*_max_) value of 74% at 25 μM. Consequently, Compound **10a** was selected for further biological evaluations based on the initial screening results.

#### 2.2.2 Preferential HDAC1 Degradation and DC_50_ Value Determination for **10a**

**Figure 3:**
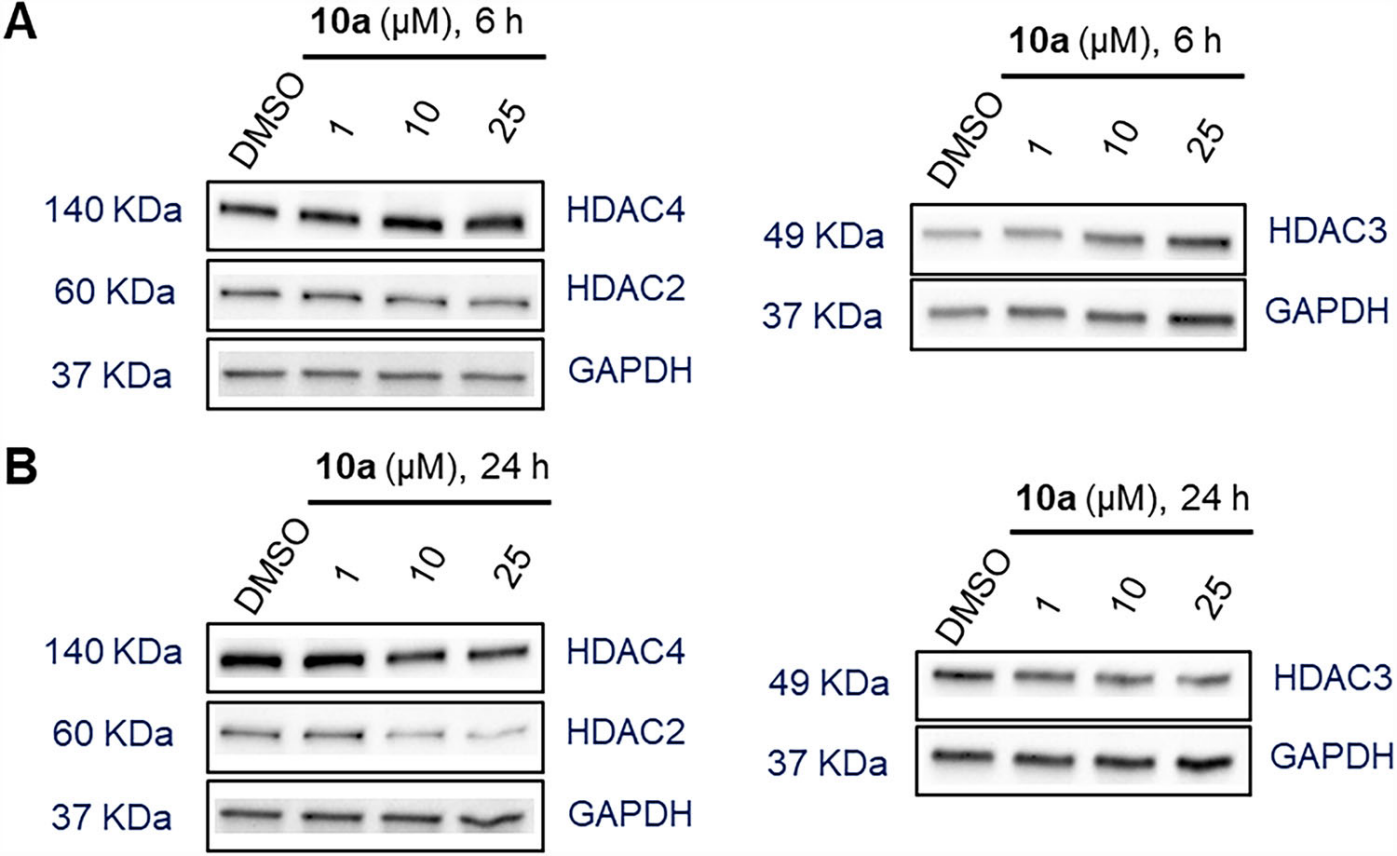
Degradation selectivity of **10a**. MM.1S cells were treated with Compound **10a** at concentrations of 1, 10, and 25 μM for 6 h (A) or for 24 h (B). DMSO was used as a vehicle control. HDAC2, HDAC3, and HDAC4 levels were detected by immunoblot analysis. GAPDH was used as a loading control. Representative images from a total of *n* = 2 replicates.

Subsequently, degrader **10a** was investigated at various concentrations (1, 10, and 25 µM) for degradation effects on different HDAC isoforms, including HDAC2, HDAC3, and HDAC4. As shown in Figure 3A and Supporting Information S2: Figure S3A, none of the used concentrations resulted in the degradation of HDAC2, HDAC3, or HDAC4 when MM.1S cells were treated with **10a** for 6 h. Meanwhile, treatment with **10a** for 24 h had minimal impact on HDAC3 and HDAC4 protein levels, whereas HDAC2 levels were slightly affected (Figure 3B and Supporting Information S2: Figure S3B). In detail, **10a** induced a modest degradation of HDAC2 (*D*_max_ **=** 46% at 25 μM), which may be attributed to the high structural similarity between HDAC1 and HDAC2, particularly in their catalytic domains [16]. In summary, the results presented above clearly confirm that **10a** exhibits potent degradation activity and high preference for HDAC1 among the various tested HDAC isoforms.

Following the observation that Compound **10a** preferentially induced degradation of HDAC1 in MM.1S cells, we proceeded to determine its half‐maximal degradation concentration (DC_50_) for HDAC1 (Supporting Information S2: Figure S4). When MM.1S cells were treated with increasing concentrations of **10a** for 24 h, HDAC1 levels gradually decreased in a dose‐dependent manner (DC_50_ = 8.8 ± 4.4 μM).

#### 2.2.3 Antiproliferative Activity, HDAC Enzyme Inhibition of **10a**, and Cellular Target Engagement Studies

Building on the results obtained from western blot analysis, we subsequently assessed **10a** for its antiproliferative activity in MM.1S cells and for its inhibitory activity against HDAC1 and HDAC6. As shown in Table 1, Compound **10a** exhibited substantial antiproliferative activity (half‐maximal inhibitory concentration, IC_50_ **=** 6.48 μM) against MM.1S cells. The possible mechanism of antiproliferative activities is the induction of HDAC1 degradation by **10a**, which supports the critical role of HDAC1 as one of the most relevant isoforms in cancer. Regarding HDAC inhibition, **10a** displayed notable inhibitory activity against HDAC1 (IC_50_ **=** 0.017 μM) and HDAC6 (IC_50_ **=** 0.027 μM).

**Table 1:** Antiproliferative activities against MM.1S cells and HDAC1 and 6 enzyme inhibition of **10a**.

	IC_50_ (μM)	IC_50_ (μM)	IC_50_ (μM)
Compound	Cell viability^a^	HDAC1^b^	HDAC6^b^
**10a**	6.48 ± 1.18	0.017 ± 0.001	0.027 ± 0.008
Ricolinostat	2.59 ± 0.27	n.d.	n.d.
Vorinostat	0.79 ± 0.13	0.064 ± 0.012	0.030 ± 0.017

^a^ *n* = 3 biologically independent replicates. MM.1S cells were treated with the indicated compounds in increasing concentration for 72 h followed by a CellTiter‐Glo cell viability assay.

^b^ *n* = 2 biologically independent replicates. In all cases, mean ± standard deviation is shown. n.d. = not determined.

Based on the HDAC6 inhibition data, the lack of efficient HDAC6 degradation by **10a** (Figure 2) is surprising. However, efficient TPD depends not only on binding affinity and ternary complex formation but also on subcellular localization. Since DCAF16 is predominantly localized in the nucleus [17], a DCAF16‐recruiting degrader is more likely to target nuclear HDACs, such as HDAC1, rather than cytoplasmic isoforms like HDAC6.

**Figure 4:**
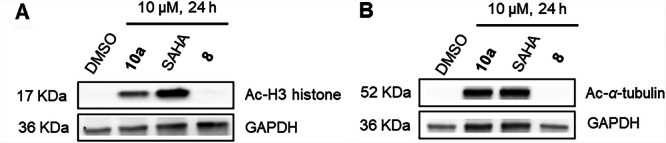
Cellular target engagement by **10a,** vorinostat (SAHA), and **8**. Immunoblot analysis of acetylated histone H3 (A) and *α*‐tubulin (B) in MM.1S cell lysates after treatment with the indicated compounds (10 µM) or vehicle (DMSO) for 24 h. Representative images from a total of *n* = 2 replicates.

To assess the cellular target engagement of degrader **10a** of HDACs in MM.1S cells, we conducted western blot experiments to analyze the levels of acetylated histone H3 (an indicator of reduced HDAC1‐3 activity) and acetylated *α*‐tubulin (a marker of decreased HDAC6 activity) using vorinostat as a control. Consistent with the results of the biochemical HDAC inhibition assays, degrader **10a** resulted in a pronounced upregulation of acetylated H3 histone and acetylated *α*‐tubulin after 24‐h treatment, indicating that **10a** exhibited significant inhibition or degradation of both HDAC1 and HDAC6 in MM.1S cells. As expected, the DCAF16‐targeting chemical handle **8** exhibited no effects on the levels of acetylated histone H3 and acetylated *α*‐tubulin (Figure 4). Interestingly, Compound **10a** induced hyperacetylation of *α*‐tubulin comparable to its parent inhibitor, vorinostat, thereby indicating sufficient cellular permeability. However, its effect on histone H3 acetylation was markedly lower than that of vorinostat. This discrepancy may suggest reduced nuclear permeability of **10a**, which could also account for its diminished activity in the viability assays (Table 1).

#### 2.2.4 Negative Control Compound and HDAC1‐3 Inhibition

**Figure 5:**
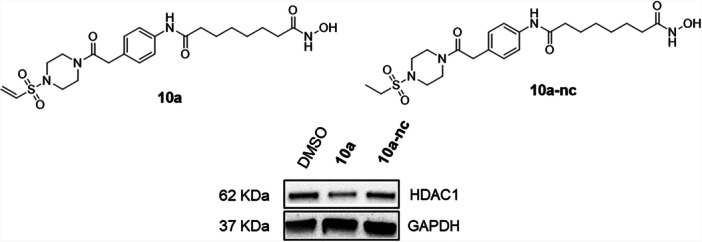
Degradation of HDAC1 mediated by **10a** and negative control **10a‐nc**. MM.1S cells were treated with Compounds **10a** and **10a‐nc** at a concentration of 25 μM for 24 h. DMSO was used as a vehicle control. HDAC1 levels were detected by immunoblot analysis. GAPDH was used as the loading control. Representative images from a total of *n* = 2 replicates.

To assess the functional relevance of the covalent vinylsulfonyl piperazine moiety in mediating HDAC1 degradation, we synthesized a noncovalent control compound, designated **10a‐nc**, in which the vinylsulfonyl warhead was replaced by an unreactive ethylsulfonyl analog (see Supporting Information S2: Scheme S1, for synthetic details). Biochemical HDAC inhibition assays showed that **10a‐nc**, while less potent than **10a**, effectively inhibits HDAC1–3 with submicromolar IC_50_ values (Table 2). The subsequent immunoblot analysis revealed that only **10a**, and not **10a‐nc**, induced a reduction of HDAC1 protein levels (Figure 5). These findings underscore the essential role of the electrophilic vinylsulfonyl piperazine handle for the knockdown of HDAC1.

**Table 2:** HDAC1–3 enzyme inhibition of **10a** and **10a‐nc**.

	IC_50_ (μM)	IC_50_ (μM)	IC_50_ (μM)
Compound	HDAC1^a^	HDAC2^a^	HDAC3^a^
**10a**	0.017 ± 0.001	0.088 ± 0.007	0.051 ± 0.004
**10a‐nc**	0.229 ± 0.004	0.423 ± 0.063	0.323 ± 0.022
Vorinostat	0.064 ± 0.012	0.203 ± 0.055	0.129 ± 0.002

^a^ *n* = 2 biologically independent replicates, mean ± standard deviation is shown.

#### 2.2.5 Apoptosis Induction in MM.1S Cells After **10a** Treatment

**Figure 6:**
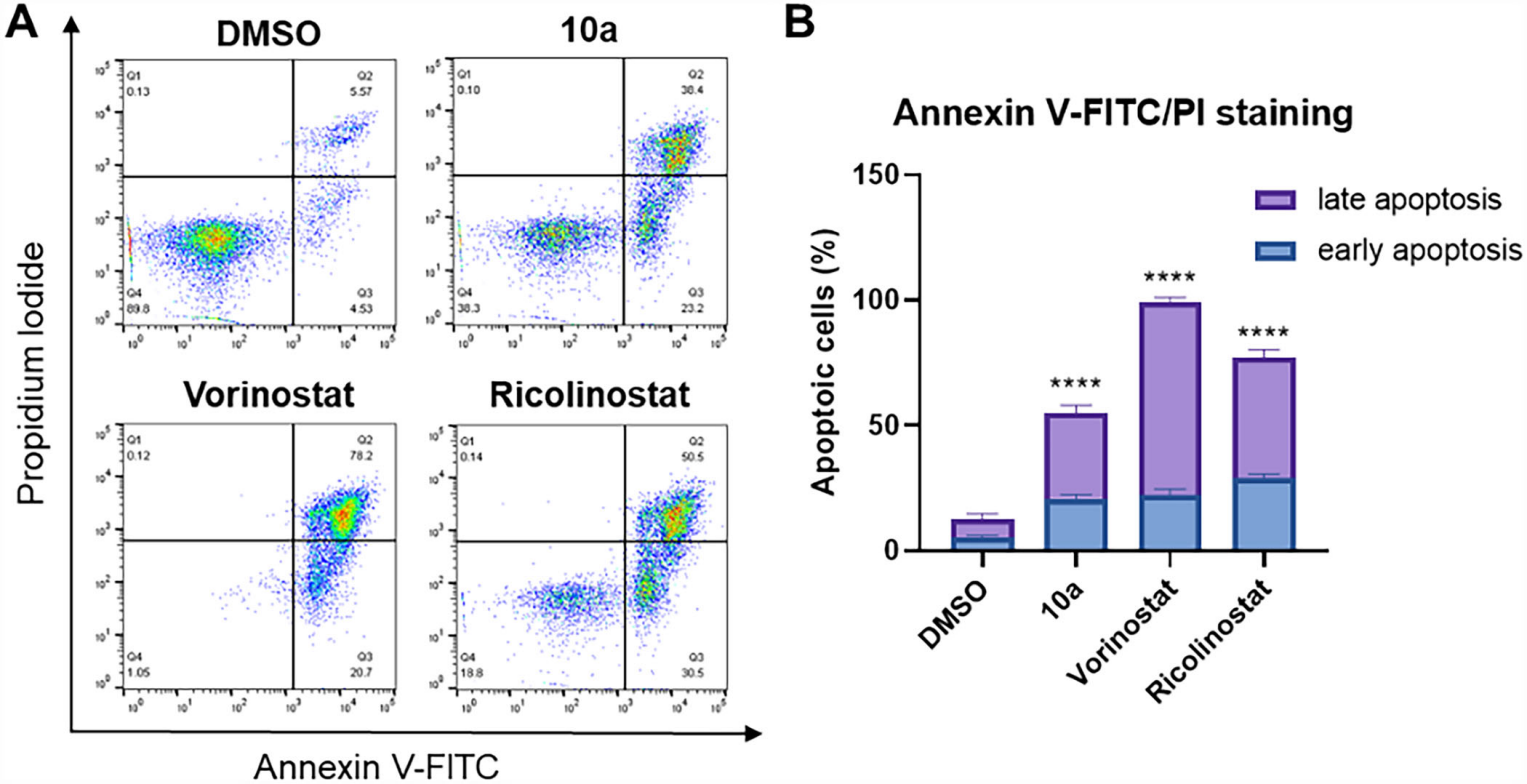
(A) Flow cytometry analysis of MM.1S cells stained with annexin V‐FITC/PI after incubation with **10a** (10 μM), vorinostat (5 μM), ricolinostat (5 μM), or DMSO for 48 h. Representative images are shown. (B) Quantification of early and late apoptotic cells. The percentage of cells that were annexin V‐positive but PI‐negative was considered as early apoptotic, whereas the percentage of cells that were both annexin V‐ and PI‐positive was considered as late apoptotic. Data are presented as mean ± standard deviation (*n* = 2 biological replicates, each performed in duplicates). Statistical analysis was performed by using one‐way ANOVA in GraphPad Prism 8. Statistical significance was indicated with asterisks (****p ≤ 0.0001).

Finally, following a 48‐h incubation of MM.1S cells with **10a**, along with vorinostat and ricolinostat as positive controls, apoptosis induction was evaluated using annexin V‐FITC/propidium iodide (PI) staining and flow cytometry (Figure 6). As expected, **10a** markedly increased the proportions of both early and late apoptotic cells, confirming its anticancer activity via apoptosis induction. This potency in triggering apoptosis aligned with the findings from the cell viability assays (Table 1).

## 3 Conclusion

The covalent vinylsulfonyl piperazine handle, first reported by Nomura and co‐workers, requires further optimization to improve its potency, selectivity, and pharmacokinetic properties [12]. Although the authors successfully incorporated this warhead into a range of small‐molecule degraders targeting proteins such as BRD4, CDK4, the androgen receptor, BTK, SMARCA2/4, and BCR‐ABL/c‐ABL, selectivity remained a significant challenge. Specifically, off‐target degradation was observed across the reported degraders, likely due to nonspecific interactions of the covalent handle [12]. Despite its lack of optimization, the covalent vinylsulfonyl piperazine handle presents a promising avenue for developing molecular glue‐like molecules. Although off‐target effects or nonselective HDAC inhibition cannot be ruled out as contributors to the observed phenotypic effects of **10a**, we demonstrated that the vinylsulfonyl piperazine handle's target scope can be successfully extended to HDACs.

In summary, we designed, synthesized, and characterized a new class of DCAF16‐based covalent molecular glues utilizing different ZBGs for the targeted degradation of HDACs. Western blot analysis demonstrated that the hydroxamate‐based degrader **10a** effectively reduced HDAC1 levels in MM.1S cells in a potent and preferential manner, whereas the control Compound **10a‐nc** did not affect HDAC1 levels. Subsequent cell viability assays and apoptosis induction analysis further confirmed the promising anticancer activity of **10a**. Taken together, the vinylsulfonyl piperazine handle serves as a versatile covalent warhead, enabling the conversion of the nondegradative HDAC inhibitor vorinostat into the molecular glue degrader **10a** for targeted HDAC1 degradation. This approach highlights the potential of this adaptable covalent chemical handle in drug development.

## 4 Experimental

### 4.1 Chemistry

#### 4.1.1 General

Chemicals were obtained from BLDpharm, Sigma‐Aldrich, TCI Chemicals, and abcr GmbH and used without purification. Air‐sensitive reactions were carried out under argon atmosphere utilizing standard *Schlenk* techniques. Thin‐layer chromatography (TLC) was carried out on prefabricated plates (silica gel 60, F254, Merck). Components were visualized by irradiation with ultraviolet light (254 nm). Column chromatography was carried out on silica gel (60 Å, 40−60 μm, *Acros Organics*).

Nuclear magnetic resonance (NMR) spectroscopy: Proton (^1^H) and carbon (^13^C) NMR spectra were recorded either on a Bruker AvanceDRX 500 (500 MHz ^1^H NMR, 126 MHz ^13^C NMR) or a BrukerAvance III 600 (600 MHz ^1^H NMR, 151 MHz ^13^C NMR). The chemical shifts are given in parts per million (ppm). Deuterated chloroform (CDCl_3_) and deuterated dimethyl sulfoxide (DMSO‐*d*_6_) were used as solvents.

High‐performance liquid chromatography (HPLC): A *Thermo Fisher Scientific* UltiMate 3000 UHPLC system with a Nucleodur 100−5 C18 (250 mm × 4.6 mm, *Macherey Nagel*) with a flow rate of 1 mL/min and a temperature of 25°C or a 100−5 C18 (100 mm × 3 mm, *Macherey Nagel*) with a flow rate of 0.5 mL/min and a temperature of 25°C with an appropriate gradient were used. For preparative purposes a AZURA Prep. 500/1000 gradient system with a Nucleodur 110−5 C18 HTec (250 mm × 32 mm, *Macherey Nagel*) column with 20 mL/min was used. Detection was implemented by UV absorption measurement at a wavelength of *λ* = 220 nm and *λ* = 250 nm. Bidest. H_2_O (A) and CH_3_CN (B) were used as eluents with an addition of 0.1% TFA for Eluent A. The purity of all final compounds was 95% or higher. Purity was determined via HPLC with the Nucleodur 100−5 C18 (250 mm × 4.6 mm, *Macherey Nagel*) at 250 nm.

Flash chromatography was performed on an Interchim puriFlash XS 520 Plus with a diode‐array detector (DAD) from 200 to 400 nm using prepacked silica gel cartridges (PF‐30SIHP‐F0012‐F0040) or C18 reversed‐phase cartridges (PF‐30C18HP‐F0004‐F0012).

The InChI codes of the investigated compounds, together with some biological activity data, are provided as Supporting Information S1.

#### 4.1.2 Synthesis of Methyl 2‐(4‐Aminophenyl)acetate (**2**)

Thionyl chloride (787 mg, 6.60 mmol, 1.0 eq.) was added dropwise to a solution of 4‐aminophenylacetic acid (**1**, 1000 mg, 6.60 mmol, 1.0 eq.) in anhydrous methanol (25 mL). The mixture was stirred at 80°C for 16 h. The resulting solution was cooled, and the solvent was removed under reduced pressure. The brown solid hydrochloride salt of the title compound was triturated with Et_2_O (2× 30 mL) to remove impurities. The free amine was liberated from its hydrochloride salt by the addition of aqueous NaHCO_3_, followed by extraction into CHCl_3_ (3× 30 mL). The organic layers were combined, dried over MgSO_4_, and the solvent removed to get Compound **2** as a light brown oil (1.10 g, yield 97%). ^1^H NMR (ppm, 500 MHz, DMSO‐*d*_6_): *δ* 6.90–6.88 (m, 2H), 6.51–6.48 (m, 2H), 4.94 (s, 2H), 3.58 (s, 3H), 3.43 (s, 2H). ESI‐MS m/z: 166.1, [M + H]^+^.

#### 4.1.3 Synthesis of 8‐{[4‐(2‐Methoxy‐2‐Oxoethyl)phenyl]amino}‐8‐Oxooctanoic Acid (**3**)

Compound **2** (700 mg, 4.69 mmol, 1.0 eq.), suberic anhydride (560 mg, 3.59 mmol, 0.8 eq.), and NaHCO_3_ (200 mg, 2.38 mmol, 0.5 eq.) were dissolved in anhydrous THF (25 mL) and stirred at room temperature for 16 h. Solid impurities were removed by filtration, and the solvent was removed under reduced pressure. The obtained residue was purified by flash column chromatography (C18 reversed phase, MeCN/H_2_O 5%–95%) to obtain **3** as a white solid (860 mg, 63% yield). ^1^H NMR (ppm, 600 MHz, CDCl_3_): *δ* 7.47–7.46 (m, 2H), 7.22–7.21 (m, 2H), 3.68 (s, 3H), 3.59 (s, 2H), 2.34 (q, *J* = 7.2 Hz, 4H), 1.73 (t, *J* = 7.2 Hz, 2H), 1.65 (t, *J* = 6.6 Hz, 2H), 1.39–1.36 (m, 4H). ESI‐MS m/z: 322.2, [M + H]^+^.

#### 4.1.4 General Procedure for the Synthesis of **4a–d**

To a mixture of **3** (321 mg, 1.0 mmol, 1.0 eq.) and DIPEA (522 μL, 3.0 mmol, 3.0 eq.) in anhydrous DMF (25 mL) was added HATU (570 mg, 1.5 mmol, 1.5 eq.), and the reaction mixture was stirred at room temperature for 30 min. Then *O*‐tritylhydroxylamine (275 mg, 1.0 mmol, 1.0 eq.), *tert*‐butyl (2‐aminophenyl)carbamate (312 mg, 1.5 mmol, 1.5 eq.), *tert*‐butyl 1‐propylhydrazine‐1‐carboxylate (216 mg, 1.5 mmol, 1.5 eq.), or *tert*‐butyl (2‐amino‐5‐(pyridin‐4‐yl)phenyl)carbamate (428 mg, 1.5 mmol, 1.5 eq.) was added, and the mixture was stirred at room temperature for 16 h. The reaction mixture was distilled under vacuum to remove DMF. The obtained residue was purified by flash column chromatography (C18 reversed phase, MeCN/H_2_O 5%–95%) to obtain **4a–d**.

Methyl 2‐(4‐{8‐oxo‐8‐[(trityloxy)amino]octanamido}phenyl)acetate (**4a**): Yellow solid, 163 mg, 28% yield. ^1^H NMR (ppm, 500 MHz, DMSO‐*d*_6_): *δ* 10.14 (s, 1H), 9.80 (s, 1H), 7.53–7.52 (m, 2H), 7.33–7.30 (m, 15H), 7.17–7.16 (m, 2H), 3.60 (s, 5H), 2.24 (t, *J* = 7.5 Hz, 2H), 1.78 (s, 2H), 1.52–1.46 (m, 2H), 1.19–1.14 (m, 4H), 1.00 (t, *J* = 7.5 Hz, 2H). ^13^C NMR (ppm, 126 MHz, DMSO‐*d*_6_): *δ* 174.56, 171.85, 171.28, 142.63, 138.25, 129.64, 129.11, 128.89, 127.62, 119.21, 91.88, 51.75, 36.48, 33.74, 32.12, 28.48, 25.09. ESI‐MS m/z: 577.5, [M − H]^−^.

Methyl 2‐{4‐[8‐({2‐[(*tert*‐butoxycarbonyl)amino]phenyl}amino)‐8‐oxooctanamido]phenyl}acetate (**4b**): Yellow solid, 436 mg, 85% yield. ^1^H NMR (ppm, 500 MHz, DMSO‐*d*_6_): *δ* 9.82 (s, 1H), 9.42 (s, 1H), 8.29 (s, 1H), 7.53–7.52 (m, 3H), 7.40 (d, *J* = 7.5 Hz, 1H), 7.17–7.11 (m, 3H), 7.08–7.05 (m, 1H), 3.60 (s, 5H), 2.34 (t, *J* = 7.5 Hz, 2H), 2.30 (t, *J* = 7.5 Hz, 2H), 1.63–1.57 (m, 4H), 1.45 (s, 9H), 1.35 (d, *J* = 3.0 Hz, 4H). ^13^C NMR (ppm, 126 MHz, DMSO‐*d*_6_): *δ* 171.84, 171.24, 153.20, 138.25, 131.25, 129.84, 129.63, 128.88, 125.16, 124.98, 124.02, 123.81, 119.19, 79.46, 51.75, 36.50, 36.09, 28.62, 28.49, 28.18, 25.26. ESI‐MS m/z: 510.2, [M − H]^−^.

*tert*‐Butyl 2‐(8‐{[4‐(2‐methoxy‐2‐oxoethyl)phenyl]amino}‐8‐oxooctanoyl)‐1‐propylhydrazine‐1‐carboxylate (**4c**): Light yellow oil, 421 mg, 88% yield. ^1^H NMR (ppm, 500 MHz, DMSO‐*d*_6_): *δ* 9.81 (d, *J* = 5.5 Hz, 2H), 7.52–7.51 (m, 2H), 7.17–7.15 (m, 2H), 3.60 (s, 5H), 3.25 (s, 2H), 2.28 (t, *J* = 7.5 Hz, 2H), 2.06 (t, *J* = 7.5 Hz, 2H), 1.58–1.50 (m, 4H), 1.45–1.35 (m, 11H), 1.30 (t, *J* = 7.0 Hz, 4H), 0.83 (t, *J* = 7.5 Hz, 3H). ^13^C NMR (ppm, 126 MHz, DMSO‐*d*_6_): *δ* 171.85, 171.26, 171.02, 138.23, 129.63, 128.89, 119.20, 79.44, 51.75, 50.08, 36.47, 33.21, 28.52, 28.02, 25.13, 25.00, 20.39, 11.23. ESI‐MS m/z: 476.2, [M − H]^−^.

Methyl 2‐{4‐[8‐({2‐[(*tert*‐butoxycarbonyl)amino]‐5‐(pyridin‐4‐yl)phenyl}amino)‐8‐oxooctanamido]phenyl}acetate (**4d**): Yellow oil, 533 mg, 77% yield. ^1^H NMR (ppm, 500 MHz, DMSO‐*d*_6_): *δ* 9.82 (s, 1H), 9.52 (s, 1H), 8.61 (q, *J* = 3.0 Hz, 2H), 8.51 (s, 1H), 7.88 (d, *J* = 1.5 Hz, 1H), 7.74 (d, *J* = 8.5 Hz, 1H), 7.63–7.58 (m, 3H), 7.53–7.51 (m, 2H), 7.17–7.15 (m, 2H), 3.60 (s, 5H), 2.39 (t, *J* = 7.0 Hz, 2H), 2.30 (t, *J* = 7.5 Hz, 2H), 1.65–1.60 (m, 4H), 1.47 (s, 9H), 1.37 (d, *J* = 3.0 Hz, 4H). ^13^C NMR (ppm, 126 MHz, DMSO‐*d*_6_): *δ* 172.13, 171.85, 171.26, 153.04, 150.39, 146.34, 138.25, 132.37, 129.94, 129.64, 128.89, 123.73, 123.53, 123.27, 120.89, 119.20, 79.86, 51.75, 36.51, 36.13, 28.64, 28.55, 28.17, 25.20. ESI‐MS m/z: 587.4, [M − H]^−^.

#### 4.1.5 General Procedure for the Synthesis of **5a–d**

**4a–d** (1.0 mmol, 1.0 eq.) were dissolved in THF/H_2_O (20 mL, v/v = 1:1). LiOH·H_2_O (2.0 eq.) was added, and the homogenous solution was stirred at room temperature for 2–12 h. The solvent was removed under reduced pressure and purified by flash column chromatography (C18 reversed phase, MeCN/H_2_O 5%–95%) to obtain **5a–d**.

2‐(4‐{8‐Oxo‐8‐[(trityloxy)amino]octanamido}phenyl)acetic acid (**5a**): White solid, 401 mg, 71% yield. ^1^H NMR (ppm, 600 MHz, DMSO‐*d*_6_): *δ* 10.24 (s, 1H), 9.81 (s, 1H), 7.43–7.42 (m, 2H), 7.33–7.28 (m, 15H), 7.12–7.11 (m, 2H), 3.18 (s, 2H), 2.22 (t, *J* = 7.2 Hz, 2H), 1.77 (s, 2H), 1.50–1.45 (m, 2H), 1.17 (t, *J* = 7.8 Hz, 4H), 0.99 (s, 2H). ^13^C NMR (ppm, 151 MHz, DMSO‐*d*_6_): *δ* 174.80, 171.06, 142.76, 136.76, 134.45, 129.41, 129.13, 127.61, 118.76, 91.79, 45.45, 36.47, 32.16, 28.54, 28.33, 25.18, 24.89. ESI‐MS m/z: 563.4, [M − H]^−^.

2‐{4‐[8‐({2‐[(*tert*‐Butoxycarbonyl)amino]phenyl}amino)‐8‐oxooctanamido]phenyl}acetic acid (**5b**): Yellow solid, 415 mg, 84% yield. ^1^H NMR (ppm, 500 MHz, DMSO‐*d*_6_): *δ* 7.56 (d, *J* = 8.0 Hz, 1H), 7.51 (d, *J* = 7.5 Hz, 1H), 7.43–7.42 (m, 2H), 7.13–7.11 (m, 2H), 6.96 (s, 1H), 6.89 (s, 1H), 3.20 (s, 2H), 2.26 (t, *J* = 7.5 Hz, 4H), 1.57 (q, *J* = 6.5 Hz, 4H), 1.43 (s, 9H), 1.32 (s, 4H). ^13^C NMR (ppm, 126 MHz, DMSO‐*d*_6_): *δ* 175.10, 171.70, 171.09, 154.15, 136.83, 134.38, 129.39, 123.62, 122.53, 122.25, 118.80, 78.19, 36.49, 31.47, 28.66, 28.53, 28.40, 25.56, 25.31. ESI‐MS m/z: 496.2, [M − H]^−^.

2‐(4‐{8‐[2‐(*tert*‐Butoxycarbonyl)‐2‐propylhydrazinyl]‐8‐oxooctanamido}phenyl)acetic acid (**5c**): White solid, 505 mg, 82% yield. ^1^H NMR (ppm, 500 MHz, DMSO‐*d*_6_): *δ* 9.82 (d, *J* = 4.0 Hz, 2H), 7.42–7.40 (m, 2H), 7.11–7.10 (m, 2H), 3.25 (s, 2H), 3.16 (s, 2H), 2.26 (t, *J* = 7.5 Hz, 2H), 2.05 (t, *J* = 7.5 Hz, 2H), 1.57–1.50 (m, 4H), 1.45–1.35 (m, 11H), 1.29 (s, 4H), 0.82 (t, *J* = 7.5 Hz, 3H). ^13^C NMR (ppm, 126 MHz, DMSO‐*d*_6_): *δ* 174.57, 171.02, 155.02, 136.68, 134.69, 129.36, 118.75, 79.41, 45.70, 36.45, 33.26, 28.55, 28.05, 25.22, 25.07, 20.43, 11.26. ESI‐MS *m*/*z*: 462.3, [M − H]^−^.

2‐{4‐[8‐({2‐[(*tert*‐Butoxycarbonyl)amino]‐5‐(pyridin‐4‐yl)phenyl}amino)‐8 oxooctanamido]phenyl}acetic acid (**5d**): Brown solid, 148 mg, 26% yield. ^1^H NMR (ppm, 500 MHz, DMSO‐*d*_6_): *δ* 10.15 (s, 1H), 9.75 (s, 1H), 8.98 (s, 1H), 8.59 (q, *J* = 2.5 Hz, 2H), 7.94 (d, *J* = 1.5 Hz, 1H), 7.75 (d, *J* = 8.5 Hz, 1H), 7.62 (q, *J* = 3.0 Hz, 2H), 7.56 (dd, *J* = 2.0, 2.0 Hz, 1H), 7.40–7.39 (m, 2H), 7.11–7.09 (m, 2H), 3.16 (s, 2H), 2.34 (t, *J* = 7.5 Hz, 2H), 2.27 (t, *J* = 7.5 Hz, 2H), 1.59 (s, 4H), 1.47 (s, 9H), 1.32 (s, 4H). ^13^C NMR (ppm, 126 MHz, DMSO‐*d*_6_): *δ* 173.91, 172.23, 171.02, 153.21, 150.36, 146.48, 136.59, 134.93, 132.42, 132.05, 130.17, 129.26, 123.65, 123.16, 120.88, 118.68, 79.59, 46.02, 36.53, 36.04, 28.65, 28.50, 28.20, 25.28. ESI‐MS *m*/*z*: 573.5, [M − H]^−^.

#### 4.1.6 Synthesis of 1‐(Vinylsulfonyl)piperazine (**8**)

*tert*‐Butyl piperazine‐1‐carboxylate (**6**, 200 mg, 1.1 mmol, 1.0 eq.) was dissolved in DCM, and TEA (448 μL, 3.3 mmol, 3.0 eq.) was added at 0°C. 2‐Chloroethanesulfonyl chloride (133 μL, 1.3 mmol, 1.2 eq.) in DCM was added dropwise, and the resulting reaction mixture was stirred at room temperature overnight. The reaction was quenched with water (3 × 30 mL) and extracted with DCM (3 × 30 mL). The organic extracts were washed once with brine, dried over Na_2_SO_4_, vacuum filtered, and concentrated in vacuo. The resultant residue **7** was directly dissolved in DCM (20 mL). TFA (2 mL) was added, and the reaction mixture was stirred at room temperature for 1 h. The volatiles were removed in vacuo to give **8** for the next step without purification (light yellow oil, 179 mg, 95% yield over the two steps). ^1^H NMR (ppm, 600 MHz, DMSO‐*d*_6_): *δ* 6.91 (q, *J* = 6.0 Hz, 1H), 6.26 (d, *J* = 9.6 Hz, 1H), 6.17 (d, *J* = 16.8 Hz, 1H), 3.25 (q, *J* = 3.6 Hz, 4H), 3.21 (d, *J* = 4.8 Hz, 4H), 1.18 (t, *J* = 7.8 Hz, 1H). ESI‐MS *m*/*z*: 177.0, [M + H]^+^.

#### 4.1.7 General Procedure for the Synthesis of **9a–d**

A mixture of **5a** (564 mg, 1.0 mmol, 1.0 eq.), **5b** (497 mg, 1.0 mmol, 1.0 eq.), **5c** (463 mg, 1.0 mmol, 1.0 eq.), or **5 d** (574 mg, 1.0 mmol, 1.0 eq.) and HATU (570 mg, 1.5 mmol, 1.5 eq.) were dissolved in DMF, DIPEA (522 μL, 3.0 mmol, 3.0 eq.) was added, and the reaction mixture was allowed to stir at room temperature for 30 min. **8** (264 mg, 1.5 mmol, 1.5 eq.) dissolved in DMF (15 mL) was added dropwise, and the reaction mixture was stirred at room temperature for 16 h. Solvents were removed under vacuum and extracted with ethyl acetate (3 × 20 mL). The organic layers were dried over Na_2_SO_4_, vacuum filtered, and concentrated in vacuo. The resultant residue was purified by flash column chromatography (C18 reversed phase, MeCN/H_2_O 5%–95%) to afford **9a–d**

*N* ^1^‐(4‐{2‐Oxo‐2‐[4‐(vinylsulfonyl)piperazin‐1‐yl]ethyl}phenyl)‐*N* ^8^‐(trityloxy)octanediamide (**9a**): Yellow solid, 239 mg, 33% yield. ^1^H NMR (ppm, 600 MHz, DMSO‐*d*_6_): *δ* 10.14 (s, 1H), 9.78 (s, 1H), 7.51–7.50 (m, 2H), 7.33–7.29 (m, 15H), 7.12–7.11 (m, 2H), 6.77 (q, *J* = 6.6 Hz, 1H), 6.15 (d, *J* = 9.6 Hz, 1H), 6.09 (d, *J* = 16.2 Hz, 1H), 3.66 (s, 2H), 3.56 (s, 4H), 3.00 (s, 2H), 2.93 (s, 2H), 2.23 (t, *J* = 7.8 Hz, 2H), 1.77 (s, 2H), 1.48 (t, *J* = 7.2 Hz, 2H), 1.16 (q, *J* = 7.2 Hz, 4H), 1.00 (s, 2H). ^13^C NMR (ppm, 151 MHz, DMSO‐*d*_6_): *δ* 171.25, 170.45, 169.35, 142.63, 137.89, 132.68, 130.10, 129.79, 129.31, 129.11, 127.64, 119.23, 91.87, 45.49, 45.21, 44.96, 40.79, 36.48, 32.12, 30.83, 28.49, 28.30, 25.11, 24.81. ESI‐MS *m*/*z*: 721.2, [M − H]^−^.

*tert*‐Butyl (2‐{8‐oxo‐8‐[(4‐{2‐oxo‐2‐[4‐(vinylsulfonyl)piperazin‐1‐yl]ethyl}phenyl)amino]octanamido}phenyl)carbamate (**9b**): Light yellow solid, 155 mg, 36% yield. ^1^H NMR (ppm, 600 MHz, DMSO‐*d*_6_): *δ* 9.79 (s, 1H), 9.41 (s, 1H), 8.28 (s, 1H), 7.52–7.49 (m, 3H), 7.38 (d, *J* = 7.8 Hz, 1H), 7.13–7.04 (m, 4H), 6.76 (q, *J* = 6.6 Hz, 1H), 6.15 (d, *J* = 10.2 Hz, 1H), 6.08 (d, *J* = 16.8 Hz, 1H), 3.64 (s, 2H), 3.55 (t, *J* = 6.0 Hz, 4H), 2.99 (s, 2H), 2.92 (s, 2H), 2.33 (t, *J* = 7.2 Hz, 2H), 2.28 (t, *J* = 7.2 Hz, 2H), 1.59 (q, *J* = 7.2 Hz, 4H), 1.44 (s, 9H), 1.33 (d, *J* = 3.0 Hz, 4H). ^13^C NMR (ppm, 151 MHz, DMSO‐*d*_6_): *δ* 171.92, 171.22, 169.36, 153.21, 137.90, 132.69, 131.26, 130.10, 129.80, 129.31, 125.19, 125.00, 124.04, 123.83, 119.23, 79.49, 45.50, 45.21, 44.97, 40.80, 38.98, 36.51, 36.09, 28.65, 28.50, 28.20, 25.27. ESI‐MS *m*/*z*: 654.2, [M − H]^−^.

*tert*‐Butyl 2‐{8‐oxo‐8‐[(4‐{2‐oxo‐2‐[4‐(vinylsulfonyl)piperazin‐1‐yl]ethyl}phenyl)amino]octanoyl}‐1‐propylhydrazine‐1‐carboxylate (**9c**): Light yellow solid, 218 mg, 47% yield. ^1^H NMR (ppm, 600 MHz, DMSO‐*d*_6_): *δ* 9.83 (s, 1H), 9.79 (s, 1H), 7.51–7.49 (m, 2H), 7.12–7.10 (m, 2H), 6.78 (q, *J* = 6.6 Hz, 1H), 6.16 (d, *J* = 10.2 Hz, 1H), 6.09 (d, *J* = 16.8 Hz, 1H), 3.66 (s, 2H), 5.56 (t, *J* = 6.0 Hz, 4H), 3.25 (s, 2H), 3.00 (s, 2H), 2.93 (s, 2H), 2.27 (t, *J* = 7.2 Hz, 2H), 2.05 (t, *J* = 7.2 Hz, 2H), 1.58–1.51 (m, 4H), 1.43–1.29 (m, 15H), 0.83 (t, *J* = 7.8 Hz, 3H). ^13^C NMR (ppm, 151 MHz, DMSO‐*d*_6_): *δ* 171.23, 171.00, 169.35, 155.00, 137.89, 132.69, 130.11, 129.79, 129.31, 119.24, 79.46, 50.09, 45.50, 45.21, 44.97, 40.80, 38.99, 36.49, 33.22, 28.54, 28.03, 25.16, 25.02, 20.41, 11.25. ESI‐MS *m*/*z*: 620.3, [M − H]^−^.

*tert*‐Butyl (2‐{8‐oxo‐8‐[(4‐{2‐oxo‐2‐[4‐(vinylsulfonyl)piperazin‐1‐yl]ethyl}phenyl)amino]octanamido}‐4‐(pyridin‐4‐yl)phenyl)carbamate (**9d**): Yellow solid, 282 mg, 39% yield. ^1^H NMR (ppm, 500 MHz, DMSO‐*d*_6_): *δ* 9.79 (s, 1H), 9.51 (s, 1H), 8.59 (q, *J* = 3.0 Hz, 2H), 8.50 (s, 1H), 7.86 (d, *J* = 2.0 Hz, 1H), 7.72 (d, *J* = 8.5 Hz, 1H), 7.62–7.57 (m, 3H), 7.50–7.48 (m, 2H), 7.11–7.09 (m, 2H), 6.76 (q, *J* = 6.0 Hz, 1H), 6.14 (d, *J* = 10.0 Hz, 1H), 6.07 (d, *J* = 16.5 Hz, 1H), 3.64 (s, 2H), 3.55 (t, *J* = 4.5 Hz, 4H), 2.98 (d, *J* = 4.0 Hz, 2H), 2.93 (d, *J* = 4.5 Hz, 2H), 2.38 (t, *J* = 7.0 Hz, 2H), 2.29 (t, *J* = 7.5 Hz, 2H), 1.64–1.59 (m, 4H), 1.46 (s, 9H), 1.35 (t, *J* = 3.0 Hz, 4H). ^13^C NMR (ppm, 126 MHz, DMSO‐*d*_6_): *δ* 172.13, 171.22, 169.35, 153.04, 150.39, 146.34, 137.89, 132.70, 132.37, 130.10, 129.94, 129.76, 129.30, 123.74, 123.54, 123.27, 120.90, 119.23, 79.86, 45.48, 45.20, 44.96, 40.79, 38.97, 36.51, 36.12, 28.65, 28.54, 28.18, 25.21. ESI‐MS *m*/*z*: 731.8, [M − H]^−^.

#### 4.1.8 General Procedure for the Synthesis of **10a–d**

**9a–d** were dissolved in DCM (10 mL) and triisopropylsilane (TIPS, 0.5 mL). TFA (1 mL) was added dropwise, and the reaction mixture was stirred at room temperature for 1 h. Solvents were removed in vacuo, and the residue was purified by flash column chromatography (C18 reversed phase, MeCN/H_2_O 5%–95%) to give **10a–d**.

*N* ^1^‐Hydroxy‐*N* ^8^‐(4‐{2‐oxo‐2‐[4‐(vinylsulfonyl)piperazin‐1‐yl]ethyl}phenyl)octanediamide (**10a**): Yellow solid, 28 mg, 25% yield. ^1^H NMR (ppm, 600 MHz, DMSO‐*d*_6_): *δ* 10.31 (s, 1H), 9.80 (s, 1H), 8.63 (s, 1H), 7.55–7.46 (m, 2H), 7.17–7.10 (m, 2H), 6.77 (q, *J* = 6.6 Hz, 1H), 6.16 (d, *J* = 10.2 Hz, 1H), 6.09 (d, *J* = 16.2 Hz, 1H), 3.65 (s, 2H), 3.56 (t, *J* = 6.0 Hz, 4H), 3.00 (s, 2H), 2.93 (s, 2H), 2.27 (t, *J* = 7.2 Hz, 2H), 1.94 (t, *J* = 7.2 Hz, 2H), 1.55 (q, *J* = 7.2 Hz, 2H), 1.48 (q, *J* = 7.2 Hz, 2H), 1.27 (s, 4H). ^13^C NMR (ppm, 151 MHz, DMSO‐*d*_6_): *δ* 171.27, 169.37, 169.26, 137.90, 132.69, 130.12, 129.81, 129.32, 119.25, 45.50, 45.22, 44.98, 40.81, 38.99, 36.49, 32.40, 28.56, 25.18. ESI‐MS *m*/*z*: 479.2, [M − H]^−^. HRMS (ESI): calcd for C_22_H_32_N_4_O_6_S, [M + H]^+^ 481.2115; found, 481.2097. HPLC: *t*_R_ = 10.82 min (98.1% purity).

*N* ^1^‐(2‐Aminophenyl)‐*N* ^8^‐(4‐{2‐oxo‐2‐[4‐(vinylsulfonyl)piperazin‐1‐yl]ethyl}phenyl)octanediamide (**10b**): White solid, 138 mg, 85% yield. ^1^H NMR (ppm, 500 MHz, DMSO‐*d*_6_): *δ* 9.81 (s, 1H), 9.57 (s, 1H), 7.53–7.49 (m, 2H), 7.23 (d, *J* = 7.5 Hz, 1H), 7.12–7.08 (m, 3H), 7.03 (d, *J* = 7.5 Hz, 1H), 6.94 (t, *J* = 7.5 Hz, 1H), 6.77 (q, *J* = 1.5 Hz, 1H), 6.16 (d, *J* = 10.0 Hz, 1H), 6.09 (d, *J* = 16.5 Hz, 1H), 3.66 (s, 2H), 3.56 (t, *J* = 4.5 Hz, 4H), 3.00 (s, 2H), 2.94 (s, 2H), 2.35 (t, *J* = 7.5 Hz, 2H), 2.29 (t, *J* = 7.5 Hz, 2H), 1.60 (q, *J* = 7.0 Hz, 4H), 1.35 (q, *J* = 3.5 Hz, 4H). ^13^C NMR (ppm, 126 MHz, DMSO‐*d*_6_): *δ* 171.91, 171.26, 169.35, 154.65, 137.89, 132.70, 130.94, 130.11, 129.77, 129.31, 126.20, 125.67, 125.59, 119.24, 45.49, 45.20, 44.96, 40.80, 38.97, 36.49, 35.77, 28.63, 28.26, 25.19. ESI‐MS *m*/*z*: 554.3, [M − H]^−^. HRMS (ESI): calcd for C_28_H_37_N_5_O_5_S, [M + H]^+^ 556.2588; found, 556.2577. HPLC: *t*_R_ = 10.82 min (99.1% purity).

8‐Oxo‐*N*‐(4‐{2‐oxo‐2‐[4‐(vinylsulfonyl)piperazin‐1‐yl]ethyl}phenyl)‐8‐(2‐propylhydrazinyl)octanamide (**10c**): White solid, 126 mg, 60% yield. ^1^H NMR (ppm, 600 MHz, DMSO‐*d*_6_): *δ* 10.88 (s, 1H), 9.82 (s, 1H), 7.51–7.50 (m, 2H), 7.12–7.10 (m, 2H), 6.78 (q, *J* = 6.6 Hz, 1H), 6.16 (d, *J* = 10.2 Hz, 1H), 6.09 (d, *J* = 16.2 Hz, 1H), 3.66 (s, 2H), 3.56 (q, *J* = 3.0 Hz, 4H), 3.00–2.94 (m, 6H), 2.28 (t, *J* = 7.2 Hz, 2H), 2.20 (t, *J* = 7.2 Hz, 2H), 1.59–1.53 (m, 6H), 1.29 (t, *J* = 3.6 Hz, 4H), 0.90 (t, *J* = 7.2 Hz, 3H). ^13^C NMR (ppm, 151 MHz, DMSO‐*d*_6_): *δ* 171.44, 171.24, 137.89, 132.70, 130.14, 129.81, 129.34, 119.24, 51.60, 45.50, 45.22, 44.97, 40.81, 38.98, 36.44, 32.98, 28.46, 25.13, 24.67, 17.96, 10.96. ESI‐MS *m*/*z*: 520.4, [M − H]^−^. HRMS (ESI): calcd for C_25_H_39_N_5_O_5_S, [M + H]^+^ 522.2745; found, 522.2750. HPLC: *t*_R_ = 10.71 min (97.5% purity).

8‐Oxo‐*N*‐(4‐{2‐oxo‐2‐[4‐(vinylsulfonyl)piperazin‐1‐yl]ethyl}phenyl)‐8‐(2‐propylhydrazinyl)octanamide (**10d**): Yellow solid, 21 mg, 40% yield. ^1^H NMR (ppm, 600 MHz, DMSO‐*d*_6_): *δ* 9.82 (s, 1H), 9.16 (s, 1H), 8.49 (q, *J* = 3.0 Hz, 2H), 7.71 (d, *J* = 1.8 Hz, 1H), 7.53–7.50 (m, 4H), 7.41 (dd, *J* = 1.8, 1.8 Hz, 1H), 7.12–7.11 (m, 2H), 6.83–6.76 (m, 2H), 6.16 (d, *J* = 9.6 Hz, 1H), 6.09 (d, *J* = 16.2 Hz, 1H), 5.27 (s, 2H), 3.66 (s, 2H), 3.56 (t, *J* = 6.0 Hz, 4H), 3.00 (s, 2H), 2.94 (s, 2H), 2.35 (t, *J* = 7.8 Hz, 2H), 2.30 (t, *J* = 7.8 Hz, 2H), 1.61 (q, *J* = 7.2 Hz, 4H), 1.36 (t, *J* = 3.0 Hz, 4H). ^13^C NMR (ppm, 151 MHz, DMSO‐*d*_6_): *δ* 171.61, 171.28, 169.36, 150.15, 147.06, 143.47, 137.90, 132.69, 130.11, 129.80, 129.32, 124.28, 123.88, 123.62, 119.82, 119.25, 116.15, 45.50, 45.21, 44.97, 40.80, 38.98, 36.52, 35.95, 28.68, 25.27. ESI‐MS *m*/*z*: 631.3, [M − H]^−^. HRMS (ESI): calcd for C_33_H_40_N_6_O_5_S, [M + H]^+^ 633.2854; found, 633.2845. HPLC: *t*_R_ = 10.68 min (98.3% purity).

#### 4.1.9 Synthesis of Negative Control *N* ^1^‐(4‐{2‐[4‐(Ethylsulfonyl)piperazin‐1‐yl]‐2‐Oxoethyl}phenyl)‐*N* ^8^‐Hydroxyoctanediamide (**10a‐nc**)

A mixture of **5a** (564 mg, 1.0 mmol, 1.0 eq.) and HATU (570 mg, 1.5 mmol, 1.5 eq.) was dissolved in DMF, DIPEA (522 μL, 3.0 mmol, 3.0 eq.) was added, and the reaction mixture was allowed to stir at room temperature for 30 min. **11** (356 mg, 2.0 mmol, 2.0 eq.) dissolved in DMF (15 mL) was added dropwise, and the reaction mixture was stirred at room temperature for 16 h. Solvents were removed under vacuum, and the residue was directly dissolved in DCM (10 mL) without purification. TFA (1 mL) was added dropwise, and the reaction mixture was stirred at room temperature for 1 h. Solvents were removed in vacuo, and the residue was purified by flash column chromatography (C18 reversed phase, MeCN/H_2_O 5%–95%) to give **10a‐nc** (light yellow oil, 61 mg, 10% yield over two steps). ^1^H NMR (ppm, 500 MHz, DMSO‐*d*_6_): *δ* 10.31 (s,1H), 9.80 (s, 1H), 7.52–7.36 (m, 2H), 7.27–7.13 (m, 2H), 3.67 (s, 2H), 3.54 (s, 4H), 3.09 (t, *J* = 19.5 Hz, 6H), 2.27 (s, 2H), 1.94 (s, 2H), 1.49 (d, *J* = 39.0 Hz, 4H), 1.19 (d, *J* = 43.0 Hz, 7H). ^13^C NMR (ppm, 126 MHz, DMSO‐*d*_6_): *δ* 171.26, 169.36, 169.25, 137.90, 130.15, 129.31, 119.25, 45.48, 45.14, 42.94, 41.25, 36.49, 32.40, 28.55, 25.18, 7.60. ESI‐MS *m*/*z*: 481.4, [M − H]^−^. HPLC: *t*_R_ = 14.19 min (98.3% purity).

### 4.2 Pharmacological/Biological Assays

#### 4.2.1 Cell Culture

The MM.1S was obtained from ATCC (Manassas, VA, USA). MM.1S cells were cultivated in RPMI 1640 medium supplemented with 10% FBS, 100 IU/mL penicillin, 0.1 mg/mL streptomycin, and 1 mM sodium pyruvate at 37°C in a 5% CO_2_ atmosphere.

#### 4.2.2 Western Blot Analysis

The MM.1S cells (3 × 10^6^ cells/mL) were seeded into cell culture flasks and, after 72 h, treated with the indicated concentration of compound or DMSO for the given time. Cell lysis was performed with Cell Extraction Buffer and the addition of Halt Protease Inhibitor Cocktail and phenylmethanesulfonyl fluoride. Protein content was determined by Pierce BCA Protein Assay Kit. Samples were denatured by Laemmli 2× Concentrate, and Precision Plus Protein Unstained Standard was used as a molecular weight marker in all cases. SDS‐PAGE was performed with 10% Mini‐PROTEAN TGX Stain‐Free Gel (Catalog# 458035, Bio‐Rad, Hercules, CA, USA) at 200 V for 50 min (Catalog# 458035, Bio‐Rad). Afterwards, proteins were transferred with the Trans‐Blot Turbo Transfer System to Immobilon‐FL PVDF membranes at 1.0 A for 30 min and incubated with 5% milk‐powder solution for 1 h at room temperature under slight agitation. Subsequently, the membranes were incubated with anti‐HDAC1 (Catalog# 5356S, Cell Signaling Technology, Denver, MA, USA), anti‐HDAC2 (Catalog# 9959S, Cell Signaling Technology, Denver, MA, USA), anti‐HDAC3 (Catalog# 85057S, Cell Signaling Technology, Denver, MA, USA), anti‐HDAC4 (Catalog# 7628S, Cell Signaling Technology, Denver, MA, USA), anti‐HDAC6 (Catalog# 7558S, Cell Signaling Technology, Denver, MA, USA), anti‐acetyl‐histone H3 (Catalog# 9677S, Cell Signaling Technology, Denver, MA, USA), anti‐acetyl‐*α*‐tubulin (Catalog# 5335, Cell Signaling Technology, Denver, MA, USA), or anti‐GAPDH (Catalog# T0004, Affinity Biosciences, Cincinnati, OH, USA) antibody solutions in 1:1000–1:20000 dilutions at room temperature under slight agitation for 1 h, then put membranes at 4°C for overnight. Incubation with HRP‐conjugated secondary anti‐mouse (Catalog# sc‐516102, Santa Cruz, Dallas, TX, USA) and anti‐rabbit (Catalog# HAF008, R&D Systems Inc., Minneapolis, MN, USA) antibody solutions was performed for 1.5 h, and membranes were developed with clarity western ECL substrate. The ChemiDoc XRS+ System was used for detection, and Image Lab Software 6.1 (Bio‐Rad, Hercules, CA, USA) for quantification [18, 19, 20].

#### 4.2.3 Celltiter‐Glo Cell Viability Assay

The MM.1S cells (2.5 × 10^3^ cells/well) were seeded in white 384‐well plates and incubated with the respective compounds at increasing concentrations. For this purpose, the dilution series was prepared at 200× concentration in DMSO and then further diluted to 10× concentration in medium. The final DMSO concentration was 0.5%. The toxicity of compounds was assessed after 72 h using the CellTiter‐Glo 2.0 cell viability assay. Luminescence was then measured, and the IC_50_ was determined by plotting dose–response curves and performing nonlinear regression using GraphPad Prism [21].

#### 4.2.4 HDAC Enzyme Inhibition Assay

For test compounds and controls, serial dilutions of the respective DMSO stock solution in assay buffer (50 mM Tris−HCl, pH 8.0, 137 mM NaCl, 2.7 mM KCl, 1.0 mM MgCl_2_·6H_2_O, and 0.1 mg/mL BSA) were prepared, and 5.0 μL of this serial dilution was transferred into OptiPlate‐96 black microplates (Revvity). A volume of 35 μL of the fluorogenic substrate ZMAL (Z‐Lys(Ac)‐AMC, 21.43 μM in assay buffer) [22] and 10 μL of enzyme solution were added. Human recombinant HDAC1 (BPS Bioscience, Catalog# 50051), HDAC2 (BPS Bioscience, Catalog# 50052), HDAC3/NcoR2 (BPS Bioscience, Catalog# 50003), or HDAC6 (BPS Bioscience, Catalog# 50006) was used. The total assay volume of 50 μL (HDAC2/3/6 max. 1% DMSO; HDAC1 max. 5% DMSO) was incubated at 37°C for 90 min. Subsequently, 50 μL of trypsin (0.4 mg/mL) in trypsin buffer (50 mM Tris−HCl, pH 8.0, 100 mM NaCl) was added, followed by an additional 30 min of incubation at 37°C. Fluorescence (excitation *λ* = 355 nm, emission *λ* = 460 nm) was measured using a FLUOstar OPTIMA microplate reader. The IC_50_ was determined by plotting dose–response curves and performing nonlinear regression using GraphPad Prism [23, 24, 25, 26].

#### 4.2.5 Annexin V/PI Assay

MM.1S cells (3 × 10^5^ cells/well) were seeded in 24‐well plates and treated with the indicated concentration of compound or DMSO for 48 h under cell culture conditions. Subsequently, cells were washed with cell staining buffer (HEPES 0.1 M, NaCl 1.4 M, CaCl_2_·3H_2_O 25 mM), resuspended in 300 µL, and 150 µL was transferred in a 96‐well plate. The staining was performed using 5 µL/well annexin V‐FITC (Catalog#640945, Biolegend, San Diego, CA, USA) and 10 µL/well PI (catalog# 421301, BioLegend, San Diego, CA, USA), incubated for 15 min and analyzed by flow cytometry (Guava easyCyte, Luminex, Austin, TX, USA) [22, 27].

## Conflicts of Interest

The authors declare no conflicts of interest.

## Supporting information

Supporting Information.

Supporting Information.

## Data Availability

The data that support the findings of this study are available from the corresponding author upon reasonable request.
