# Supplementary material for: DCAF16‐Based Covalent Molecular Glues for Targeted Protein Degradation of Histone Deacetylases
Source: Arch Pharm (Weinheim). 2025 Jul 9;358(7):e70045. doi: 10.1002/ardp.70045 (PMC12238837; doi:10.1002/ardp.70045)
Supplement: Supplementary file 1 — Supporting Information. [file ARDP-358-e70045-s001.doc]

**Supplemental Material: Novel Compounds and Biological Screening Results**

**DCAF16-based covalent molecular glues for targeted protein degradation of histone deacetylases**

Tao Sun,^1^ Shiyang Zhai,^1^ Stephan Lepper,^1^ Beate König,^1^ Mateo Malenica,^1^ Irina Honin,^1^ Finn K. Hansen^1^*

^1^Department of Pharmaceutical and Cell Biological Chemistry, Pharmaceutical Institute, University of Bonn, 53121 Bonn, Germany

*Correspondence:

Prof. Dr. Finn K. Hansen, Department of Pharmaceutical and Cell Biological Chemistry, Pharmaceutical Institute, University of Bonn, An der Immenburg 4, 53121 Bonn;

Email: finn.hansen@uni-bonn.de

| **Compound No.** | **InChI** | **IC_50_ (μM)**  **cell viability^[a]^** | **IC_50_**  **(μM)**  **HDAC1^[b]^** | **IC_50_**  **(μM)**  **HDAC2^[b]^** | **IC_50_**  **(μM)**  **HDAC3^[b]^** | **IC_50_ (μM)**  **HDAC6^[b]^** |
| --- | --- | --- | --- | --- | --- | --- |
| **10a** | InChI=1S/C22H32N4O6S/c1-2-33(31,32)26-15-13-25(14-16-26)22(29)17-18-9-11-19(12-10-18)23-20(27)7-5-3-4-6-8-21(28)24-30/h2,9-12,30H,1,3-8,13-17H2,(H,23,27)(H,24,28) | 6.48 ±  1.18 | 0.017 ± 0.001 | 0.088±0.007 | 0.051±0.004 | 0.027 ± 0.008 |
| **10b** | InChI=1S/C28H37N5O5S/c1-2-39(37,38)33-19-17-32(18-20-33)28(36)21-22-13-15-23(16-14-22)30-26(34)11-5-3-4-6-12-27(35)31-25-10-8-7-9-24(25)29/h2,7-10,13-16H,1,3-6,11-12,17-21,29H2,(H,30,34)(H,31,35) | n.d. | n.d. | n.d. | n.d. | n.d. |
| **10c** | InChI=1S/C25H39N5O5S/c1-3-15-26-28-24(32)10-8-6-5-7-9-23(31)27-22-13-11-21(12-14-22)20-25(33)29-16-18-30(19-17-29)36(34,35)4-2/h4,11-14,26H,2-3,5-10,15-20H2,1H3,(H,27,31)(H,28,32) | n.d. | n.d. | n.d. | n.d. | n.d. |
| **10d** | InChI=1S/C33H40N6O5S/c1-2-45(43,44)39-21-19-38(20-22-39)33(42)23-25-9-12-28(13-10-25)36-31(40)7-5-3-4-6-8-32(41)37-30-24-27(11-14-29(30)34)26-15-17-35-18-16-26/h2,9-18,24H,1,3-8,19-23,34H2,(H,36,40)(H,37,41) | n.d. | n.d. | n.d. | n.d. | n.d. |
| **10a-nc** | InChI=1S/C22H34N4O6S/c1-2-33(31,32)26-15-13-25(14-16-26)22(29)17-18-9-11-19(12-10-18)23-20(27)7-5-3-4-6-8-21(28)24-30/h9-12,30H,2-8,13-17H2,1H3,(H,23,27)(H,24,28) | n.d. | 0.229±0.004 | 0.423±0.063 | 0.323±0.022 | n.d. |

n.d: not determined.

**^a^CellTiter-Glo® cell viability assay**

MM.1S cells (2.5 × 10^3^ cells/well) were seeded in white 384-well plates and incubated with the respective compounds at increasing concentrations. For this purpose, the dilution series were prepared at 200× concentration in DMSO and then further diluted to 10× concentration in medium. The final DMSO concentration was 0.5%. The toxicity of compounds was assessed after 72 h using the CellTiter-Glo 2.0 cell viability assay. Luminescence was then measured, and the IC_50_ was determined by plotting dose-response curves and performing nonlinear regression using GraphPad Prism.^[1]^

**^b^HDAC enzyme inhibition assay**

For test compounds and controls, serial dilutions of the respective DMSO stock solution in assay buffer (50 mM Tris−HCl, pH 8.0, 137 mM NaCl, 2.7 mM KCl, 1.0 mM MgCl_2_·6H_2_O, 0.1 mg/mL BSA) were prepared, and 5.0 μL of this serial dilution were transferred into OptiPlate-96 black microplates (PerkinElmer). A volume of 35 μL of the fluorogenic substrate ZMAL (Z-Lys(Ac)-AMC, 21.43 μM in assay buffer)^[2]^ and 10 μL enzyme solution were added. Human recombinant HDAC1 (BPS Bioscience, Catalog# 50051), HDAC2 (BPS Bioscience, Catalog# 50052), HDAC3/NcoR2 (BPS Bioscience, Catalog# 50003), or HDAC6 (BPS Bioscience, Catalog# 50006) were used. The total assay volume of 50 μL (HDAC2/3/6 max. 1% DMSO; HDAC1 max. 5% DMSO) was incubated at 37°C for 90 min. Subsequently, 50 μL of trypsin (0.4 mg/mL) in trypsin buffer (50 mM Tris−HCl, pH 8.0, 100 mM NaCl) was added, followed by additional 30 min of incubation at 37°C. Fluorescence (excitation λ = 355 nm, emission λ = 460 nm) was measured using a FLUOstar OPTIMA microplate reader. The IC_50_ was determined by plotting dose response curves and performing nonlinear regression using GraphPad Prism.^[3-6]^

**References**

[1] Ahmed IA, Hafiz S, van Ginkel S, Pondugula SR, Abdelhaffez AS, Sayyed HG, El-Aziz EAA, Mansour MM. *Nat Prod Commun.* **2023**, *18*, 10.1177/1934578x231175323.

[2] Kraft FB, Hanl M, Feller F, Schäker-Hübner L, Hansen FK. *Pharmaceuticals (Basel).* **2023**, *16*, 356.

[3] Reßing, N.; Schliehe-Diecks, J.; Watson, P. R.; Sonnichsen, M.; Cragin, A. D.; Schöler, A.; Yang, J.; Schäker-Hübner, L.; Borkhardt, A.; Christianson, D. W.; Bhatia, S.; Hansen, F. K. *J Med Chem.* **2022**, *65*, 15457.

[4] Kraft, F. B.; Enns, J.; Honin, I.; Engelhardt, J.; Scholer, A.; Smith, S. T.; Meiler, J.; Schäker-Hübner, L.; Weindl, G.; Hansen, F. K. *Bioorg Chem* **2024**, *143*, 107072.

[5] Schäker-Hübner, L.; Warstat, R.; Ahlert, H.; Mishra, P.; Kraft, F. B.; Schliehe-Diecks, J.; Schöler, A.; Borkhardt, A.; Breit, B.; Bhatia, S.; Hügle, M.; Günther, S.; Hansen, F. K. *J Med Chem.* **2021**, *64*, 14620.

[6] König B, Watson PR, Reßing N, Cragin AD, Schäker-Hübner L, Christianson DW, Hansen FK. *J Med Chem.* **2023**, *66*, 13821.
