# Supplementary material for: DCAF16‐Based Covalent Molecular Glues for Targeted Protein Degradation of Histone Deacetylases
Source: Arch Pharm (Weinheim). 2025 Jul 9;358(7):e70045. doi: 10.1002/ardp.70045 (PMC12238837; doi:10.1002/ardp.70045)
Supplement: Supplementary file 2 — Supporting Information. [file ARDP-358-e70045-s002.pdf]

Supporting Information

DCAF16-based covalent molecular glues for targeted protein degradation of histone deacetylases

Tao Sun,[1] Shiyang Zhai,[1] Stephan Lepper,[1] Beate König,[1] Mateo Malenica,[1] Irina Honin,[1] Finn K. Hansen[1]*

[1]Department of Pharmaceutical and Cell Biological Chemistry, Pharmaceutical Institute, University of Bonn, 53121 Bonn, Germany

*Correspondence:

Prof. Dr. Finn K. Hansen, Department of Pharmaceutical and Cell Biological Chemistry, Pharmaceutical Institute, University of Bonn, An der Immenburg 4, 53121 Bonn, Germany

Email: finn.hansen@uni-bonn.de

Table of Contents

1. Supplementary Figures and Scheme S2
2. NMR data of synthesized compounds S5
3. HPLC chromatograms S22

1. Supplementary Figures and Scheme

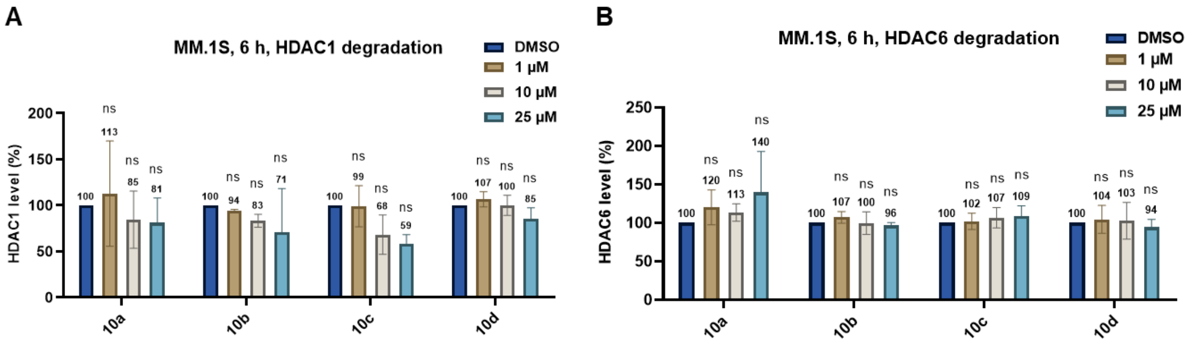

Figure S1. Densitometric analysis of HDAC1 (**A**) and HDAC6 (**B**) levels after treatment with **10a-d** for 6 h. Data from n = 2 replicates. Statistical analysis was performed by using one-way ANOVA in GraphPad Prism 8 (ns = no significance).

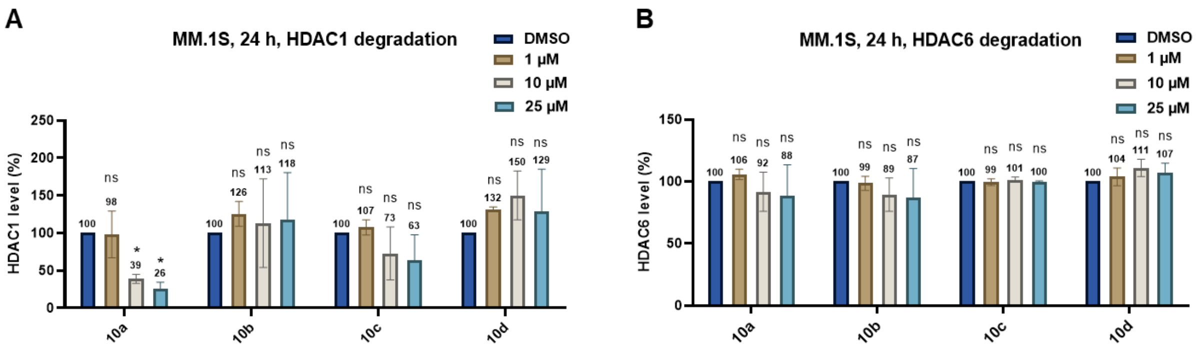

Figure S2. Densitometric analysis of HDAC1 (**A**) and HDAC6 (**B**) levels after treatment with **10a-d** for 24 h. Data from n = 2 replicates. Statistical analysis was performed by using one-way ANOVA in GraphPad Prism 8. Statistical significance was indicated with asterisks (ns = no significance; * = $p < 0.05$).

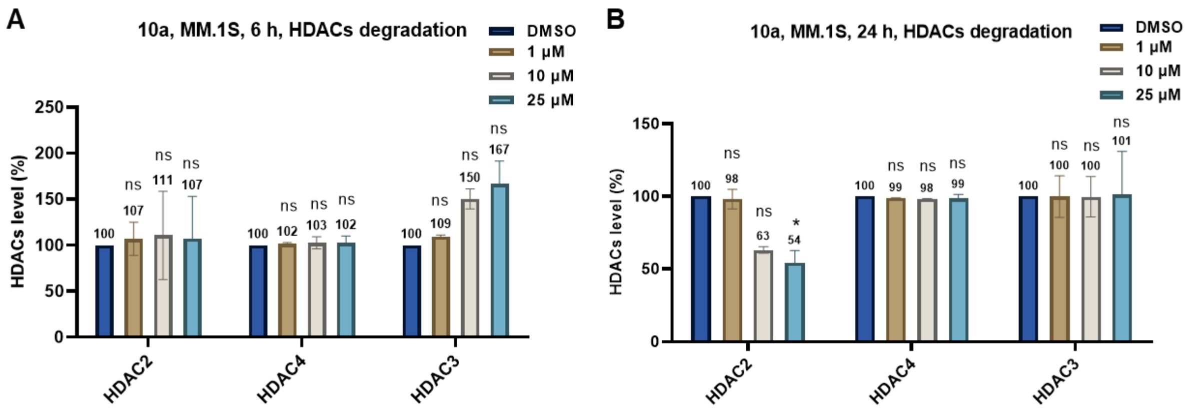

Figure S3. Densitometric analysis of HDAC2, HDAC3 and HDAC4 levels after treatment with **10a** for 6 h (**A**) or 24 h (**B**). Data from n = 2 replicates. Statistical analysis was performed by using one-way ANOVA in GraphPad Prism 8. Statistical significance was indicated with asterisks (ns = no significance; * = $p < 0.05$).

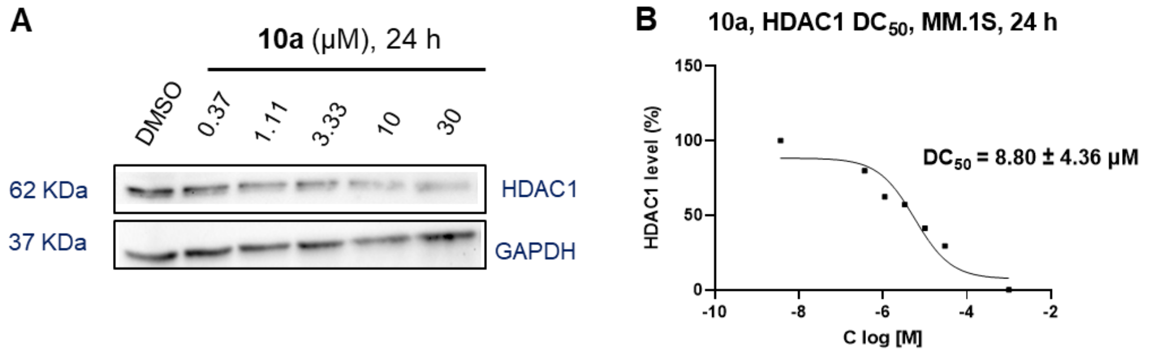

Figure S4. (**A**) Western blot analysis of HDAC1 in MM.1S cells treated for 24 h with **10a** at different concentrations ranging from 0.37 up to 30 µM. GAPDH was selected as loading control. Representative image of n = 2 replicates. (**B**) DC_{50} values were obtained by fitting D_{max} values to a variable slope response model. Representative curve of n = 2 replicates.

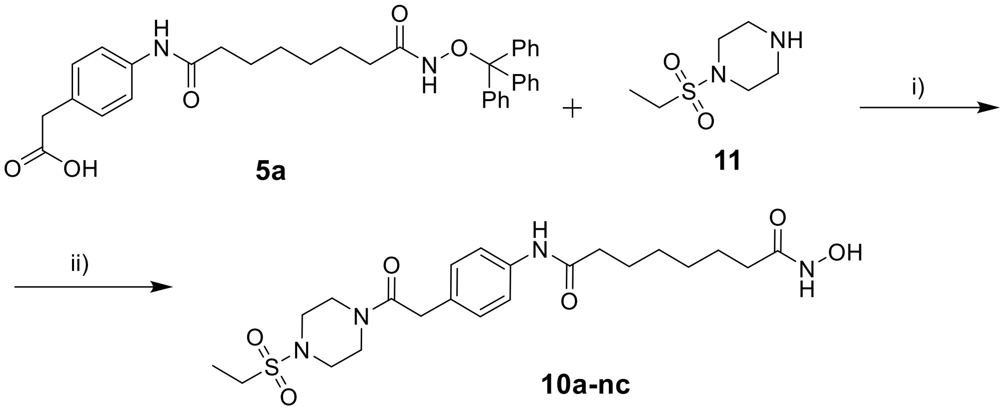

Scheme S1. Synthesis of negative control **10a-nc**. *Reagents and conditions*: **i)** HATU, DIPEA, DMF, rt, 16 h. **ii)** TFA, DCM, rt, 1 h, 10% yield (over two steps).

2. NMR data of synthesized compounds

^{1}H NMR spectrum of **4a** (500 MHz, DMSO-*d*6)

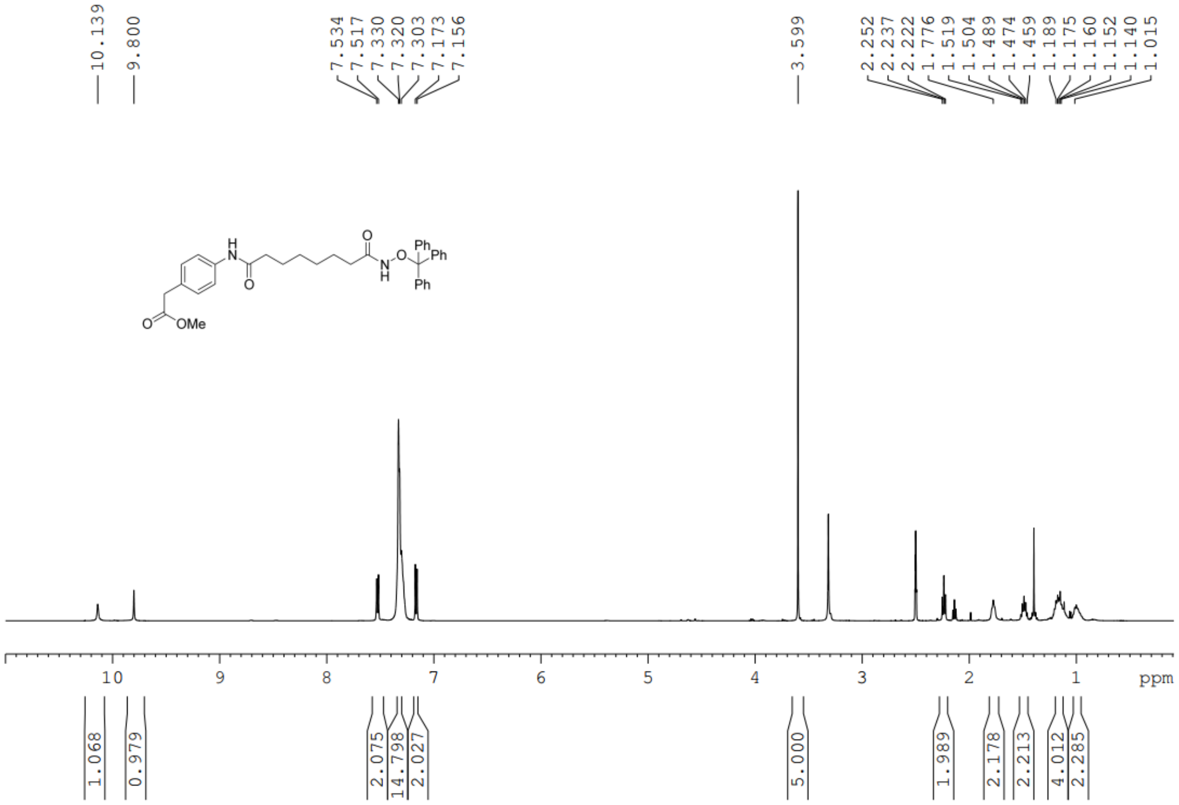

^{13}C NMR spectrum of **4a** (126 MHz, DMSO-*d*6)

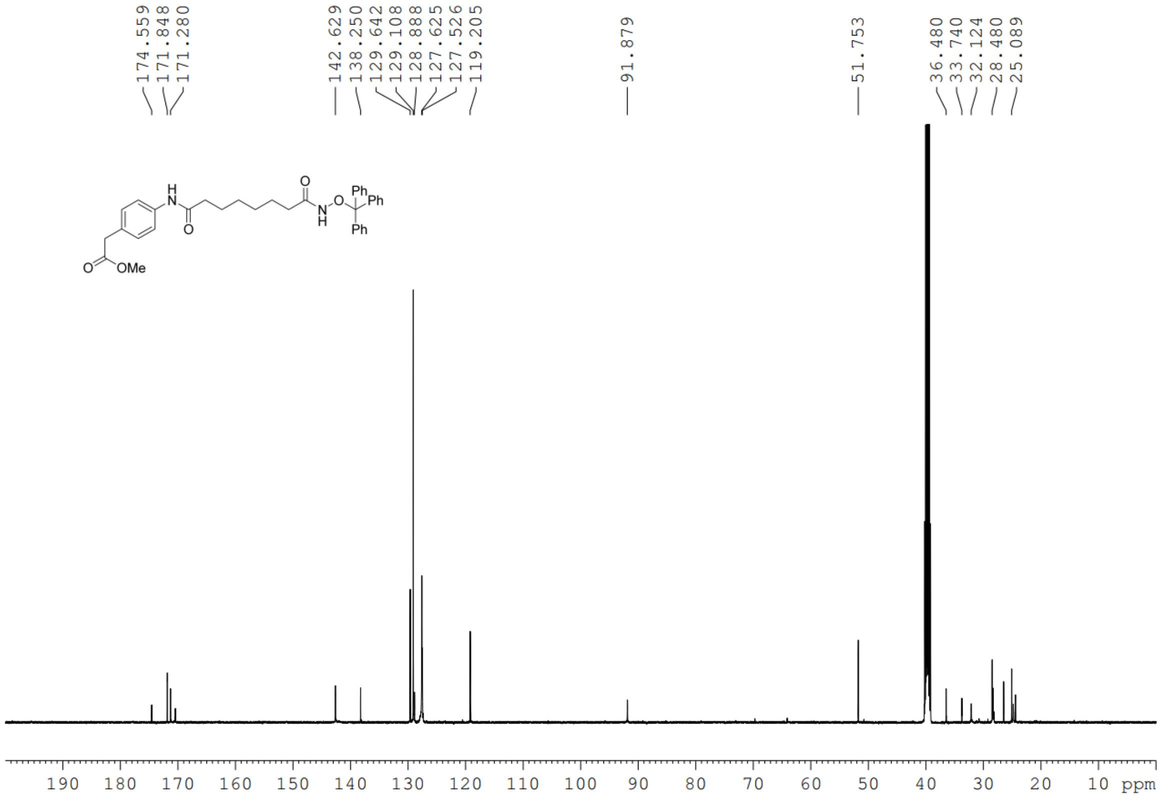

^{1}H NMR spectrum of **4b** (500 MHz, DMSO-d_6)

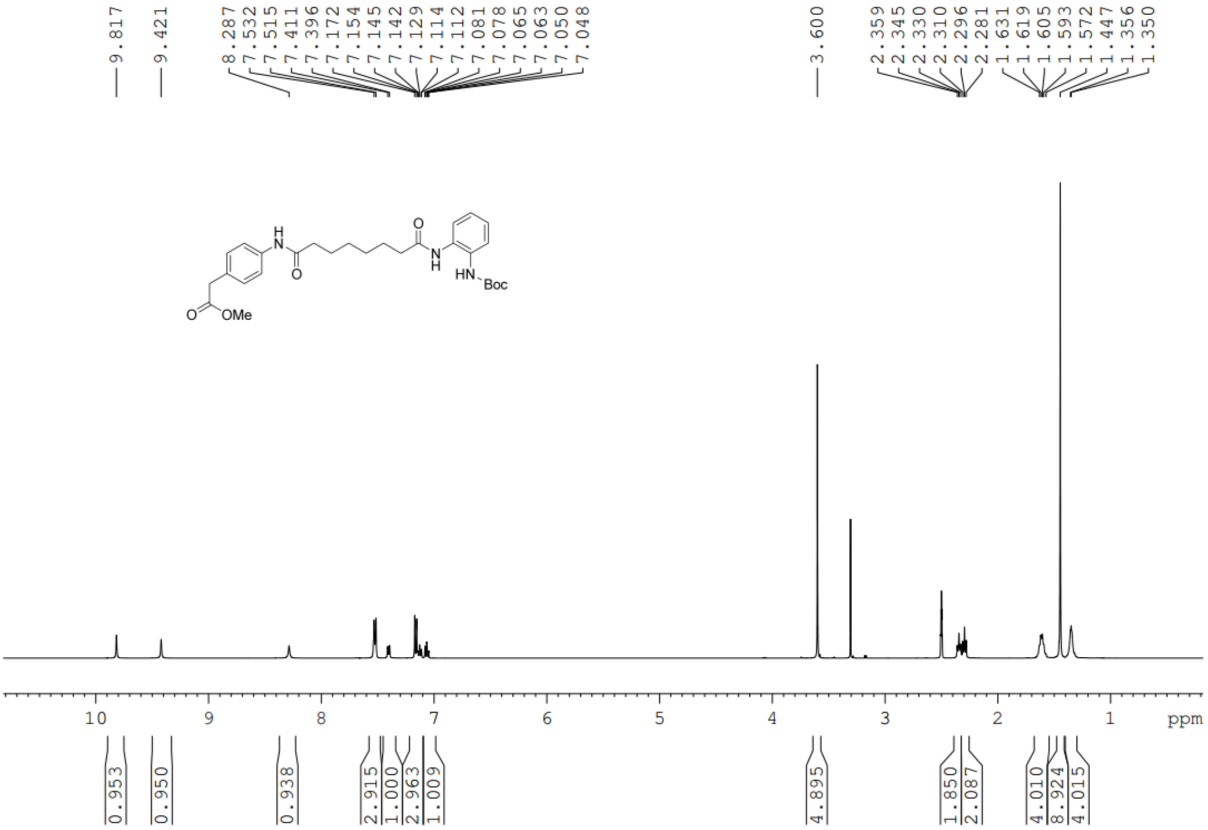

^{13}C NMR spectrum of **4b** (126 MHz, DMSO-d_6)

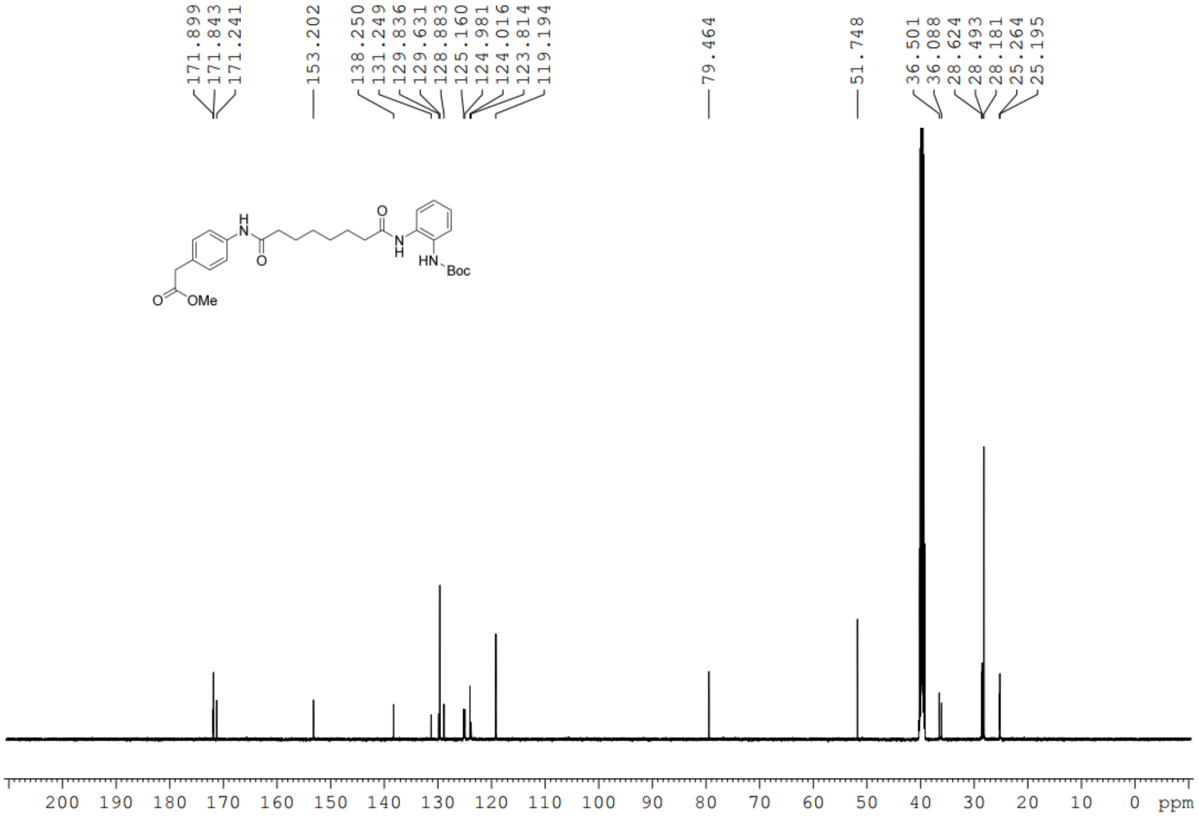

^{1}H NMR spectrum of **4c** (500 MHz, DMSO-d_6)

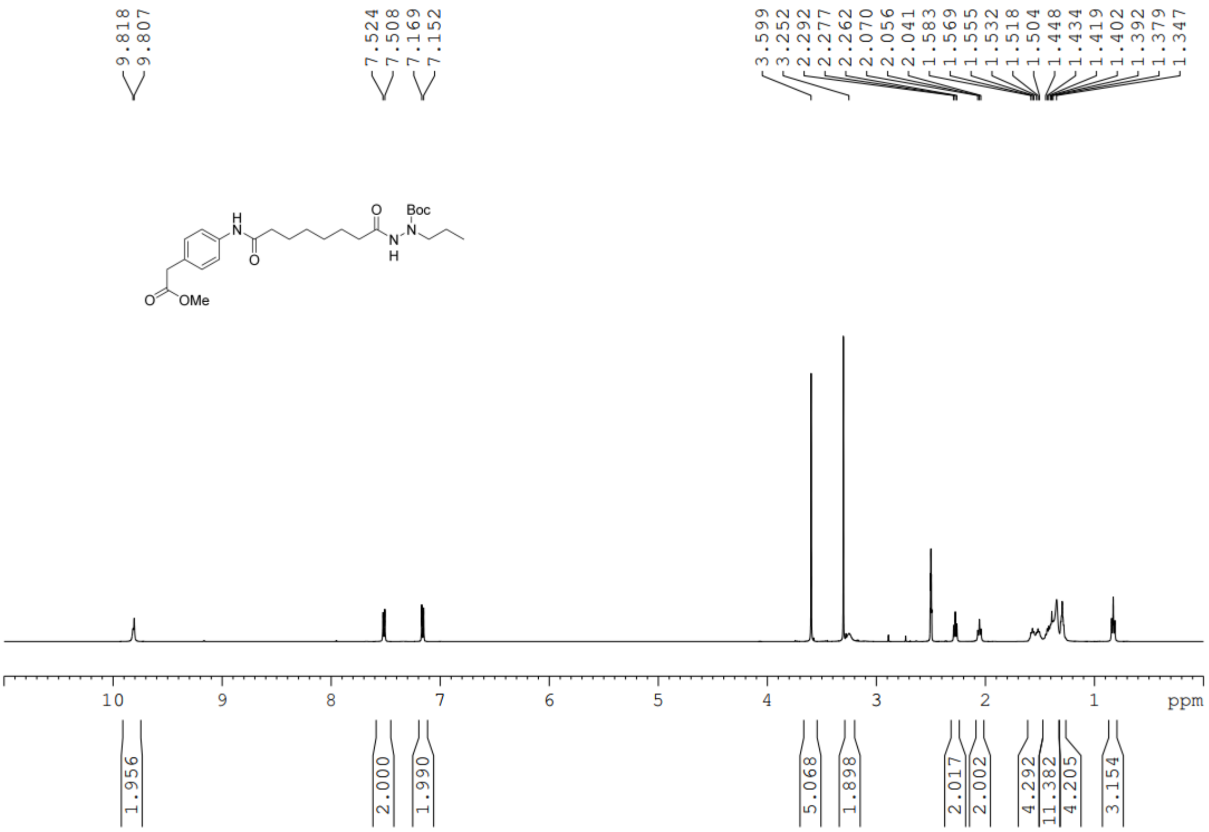

^{13}C NMR spectrum of **4c** (126 MHz, DMSO-d_6)

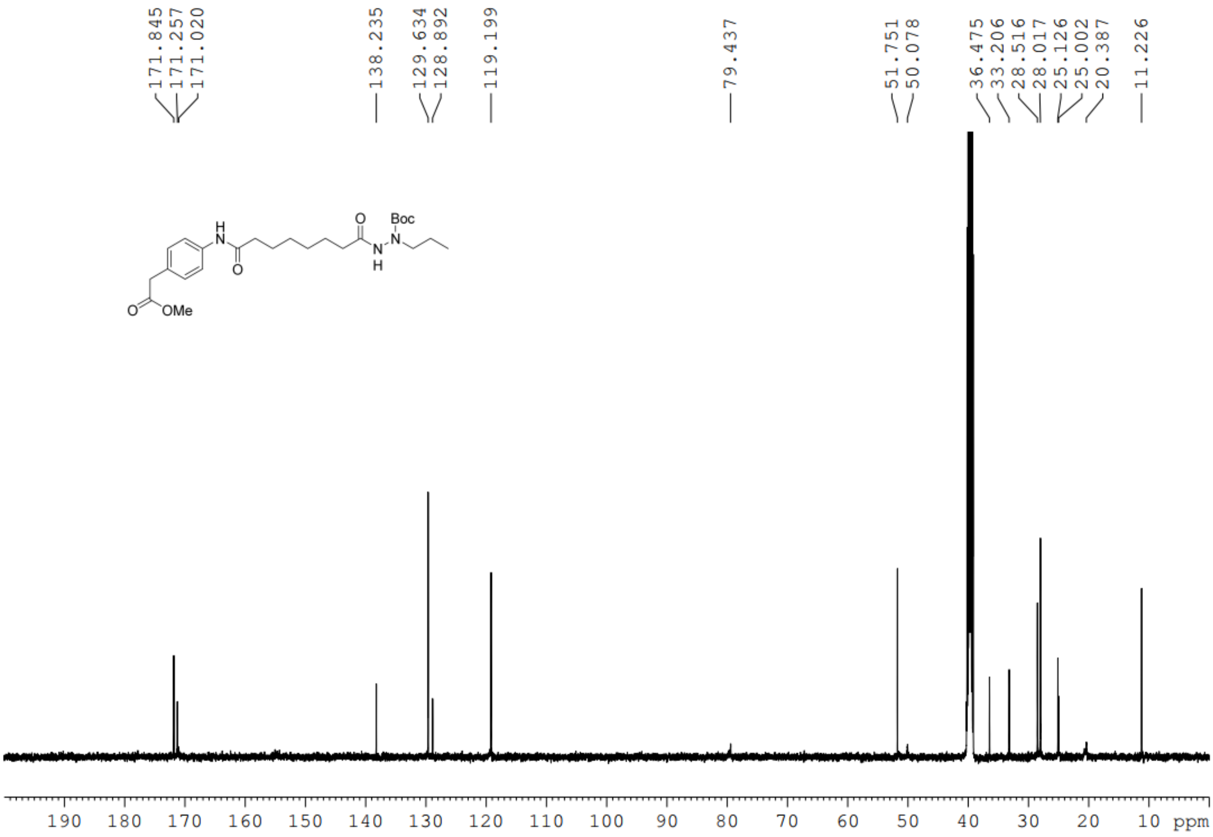

^{1}H NMR spectrum of **4d** (500 MHz, DMSO-d_6)

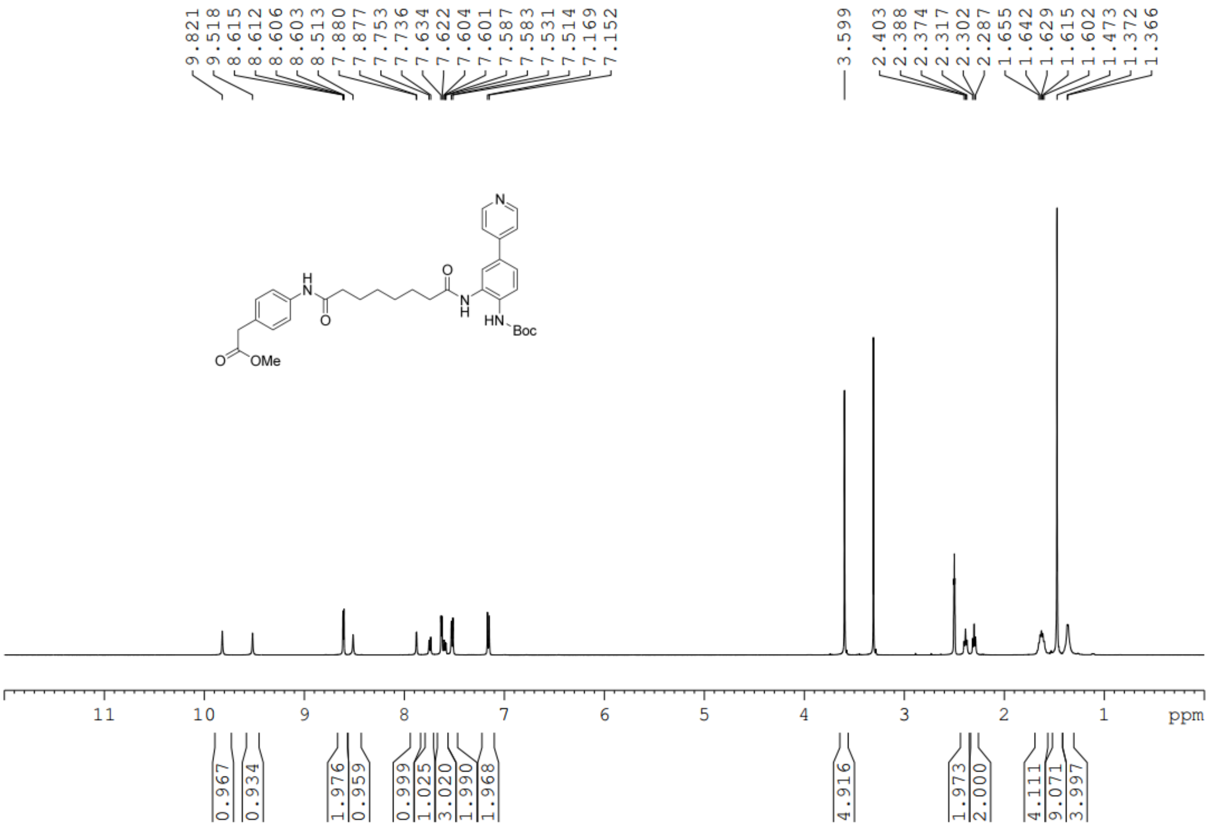

^{13}C NMR spectrum of **4d** (126 MHz, DMSO-d_6)

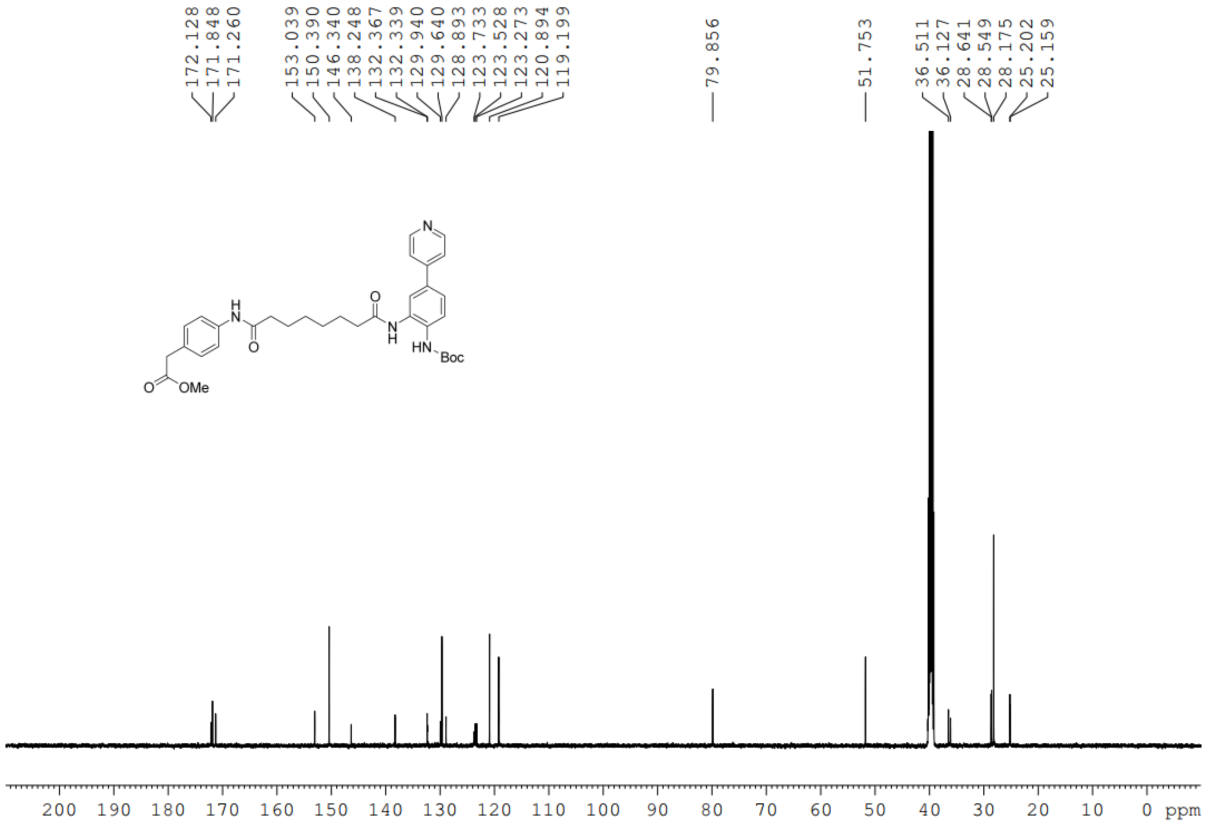

^{1}H NMR spectrum of **5a** (600 MHz, DMSO-d_6)

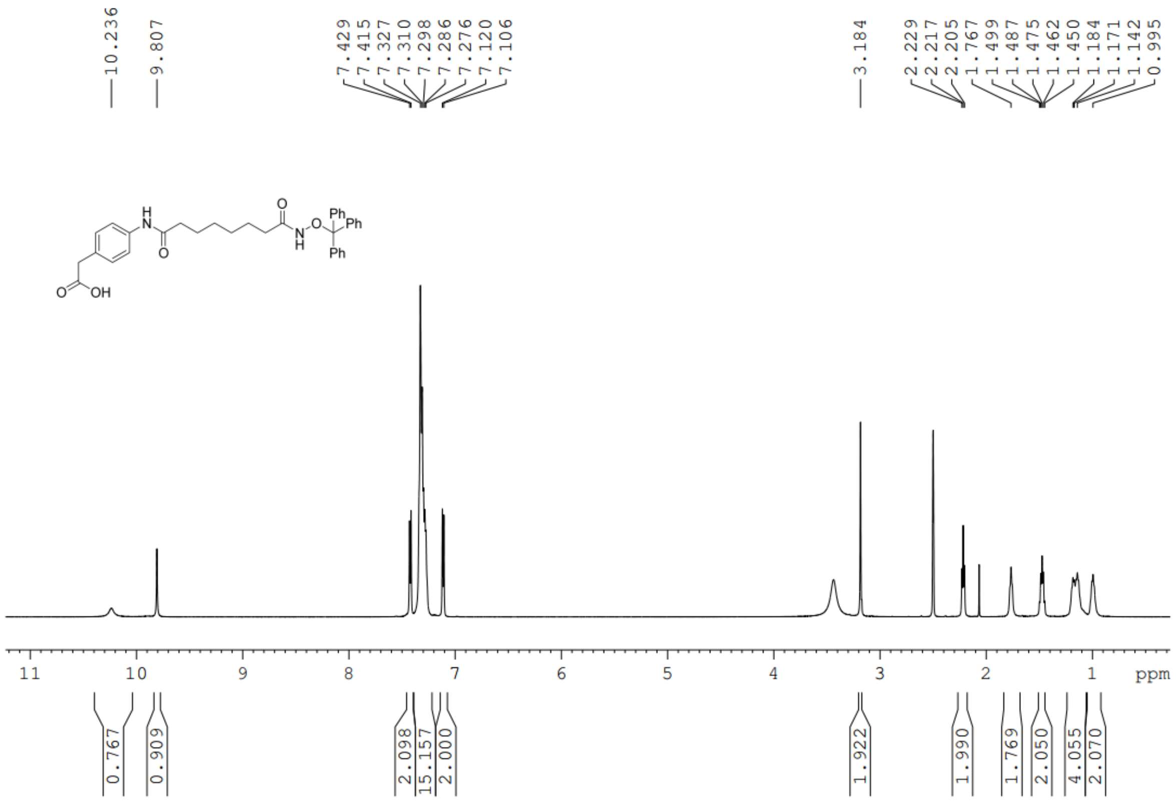

^{13}C NMR spectrum of **5a** (151 MHz, DMSO-d_6)

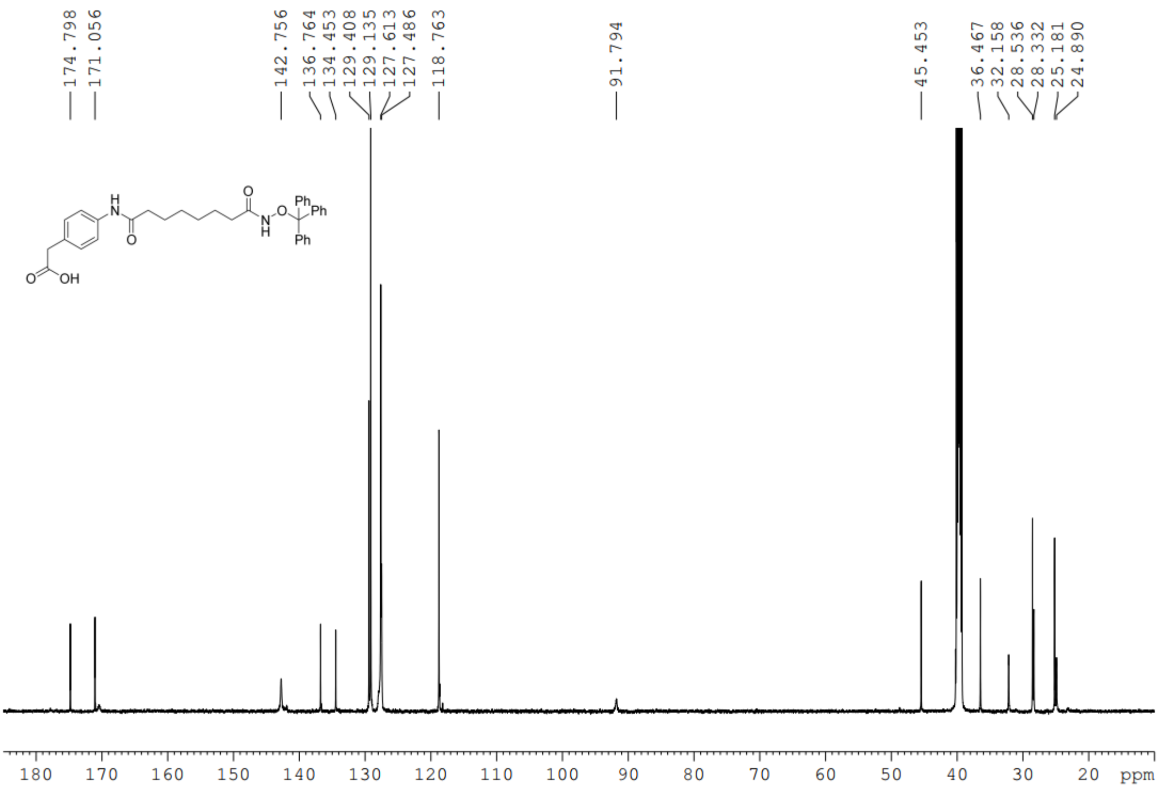

^{1}H NMR spectrum of **5b** (500 MHz, DMSO-d_6)

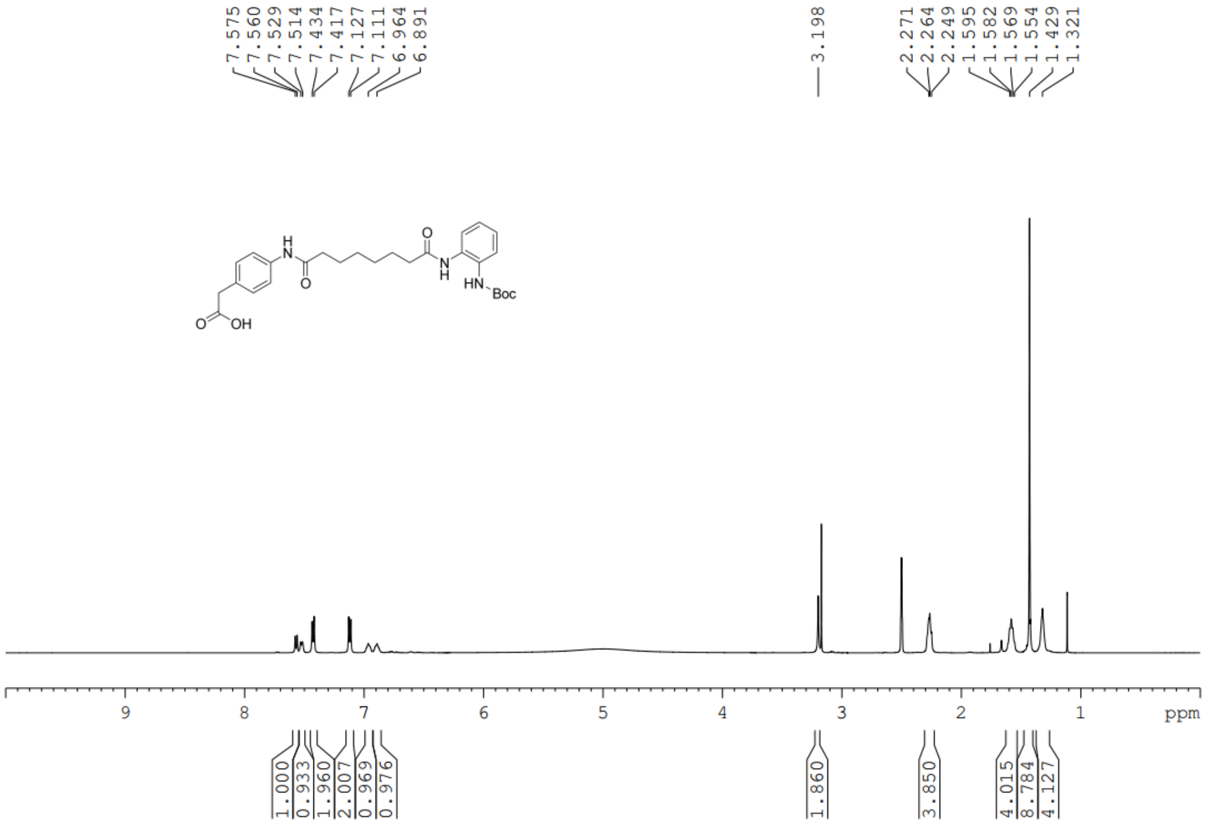

^{13}C NMR spectrum of **5b** (126 MHz, DMSO-d_6)

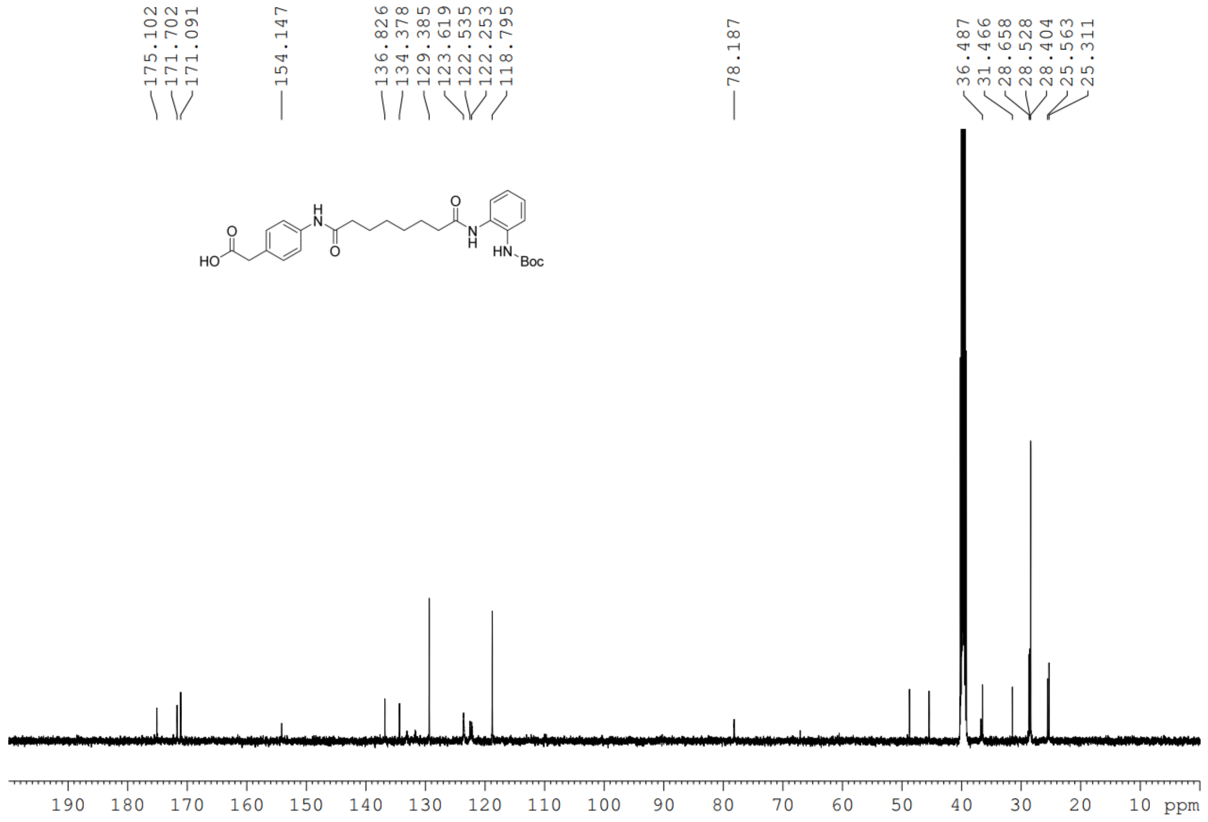

^{1}H NMR spectrum of **5c** (500 MHz, DMSO-d_6)

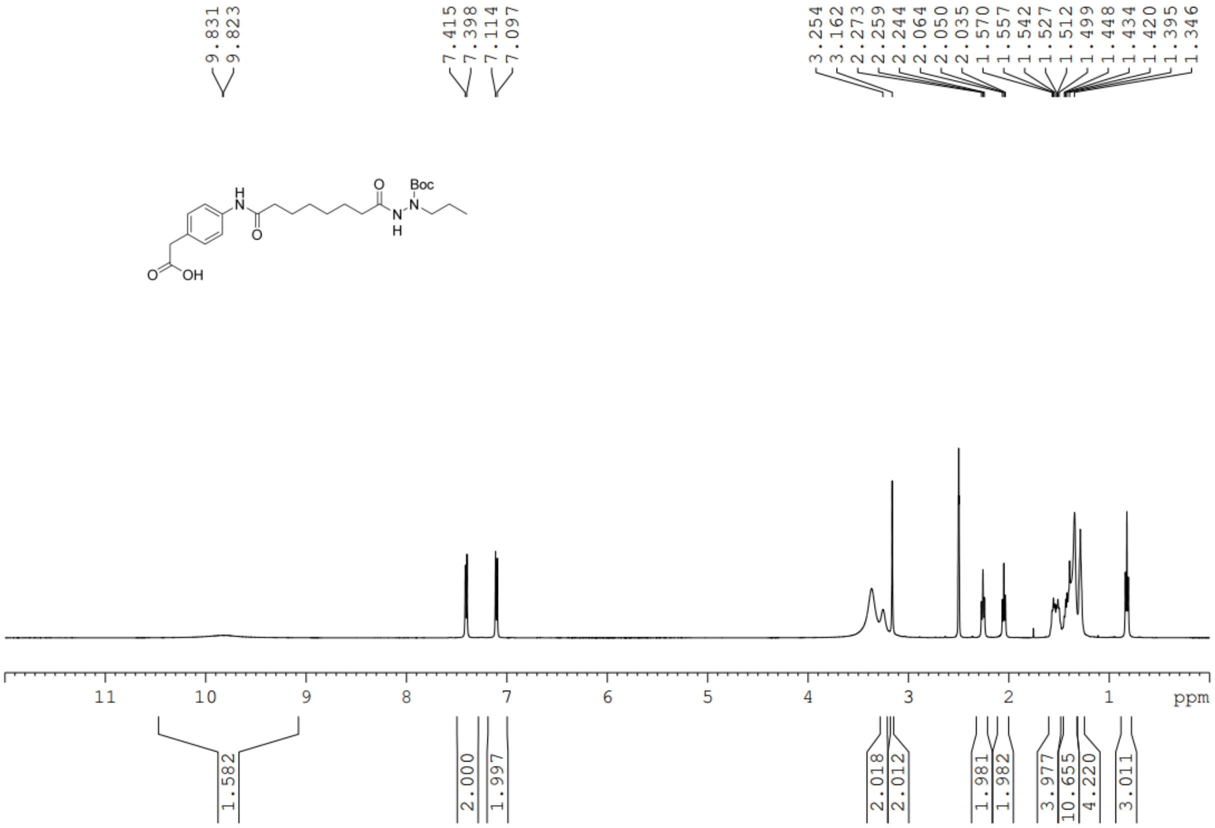

^{13}C NMR spectrum of **5c** (126 MHz, DMSO-d_6)

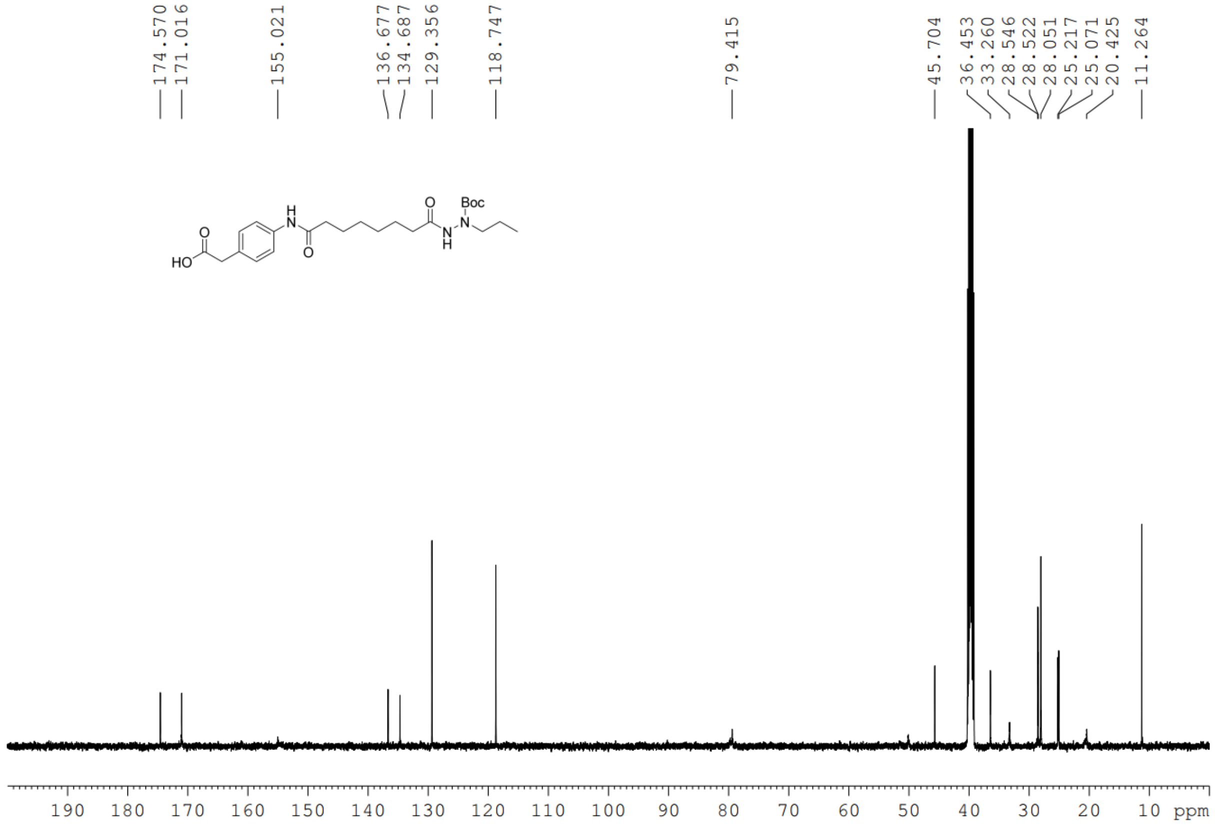

^{1}H NMR spectrum of **5d** (500 MHz, DMSO-d_6)

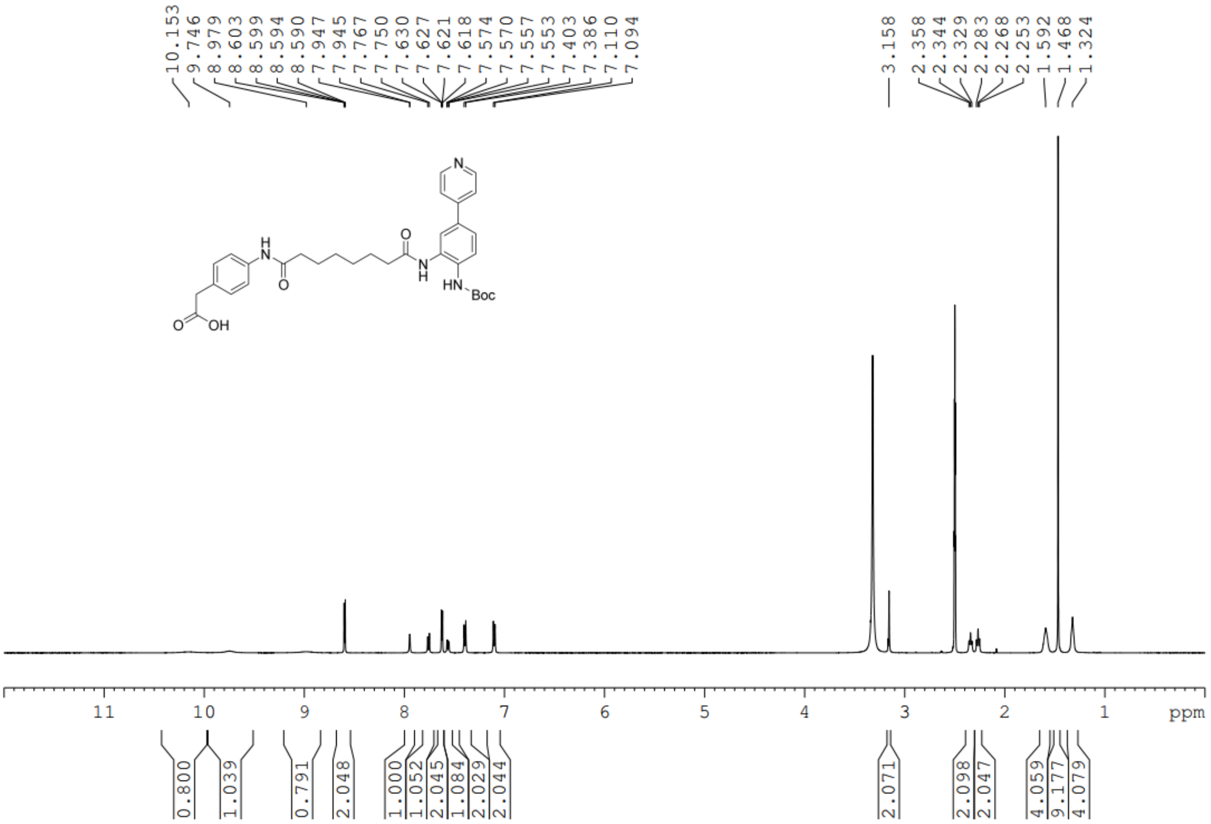

^{13}C NMR spectrum of **5d** (126 MHz, DMSO-d_6)

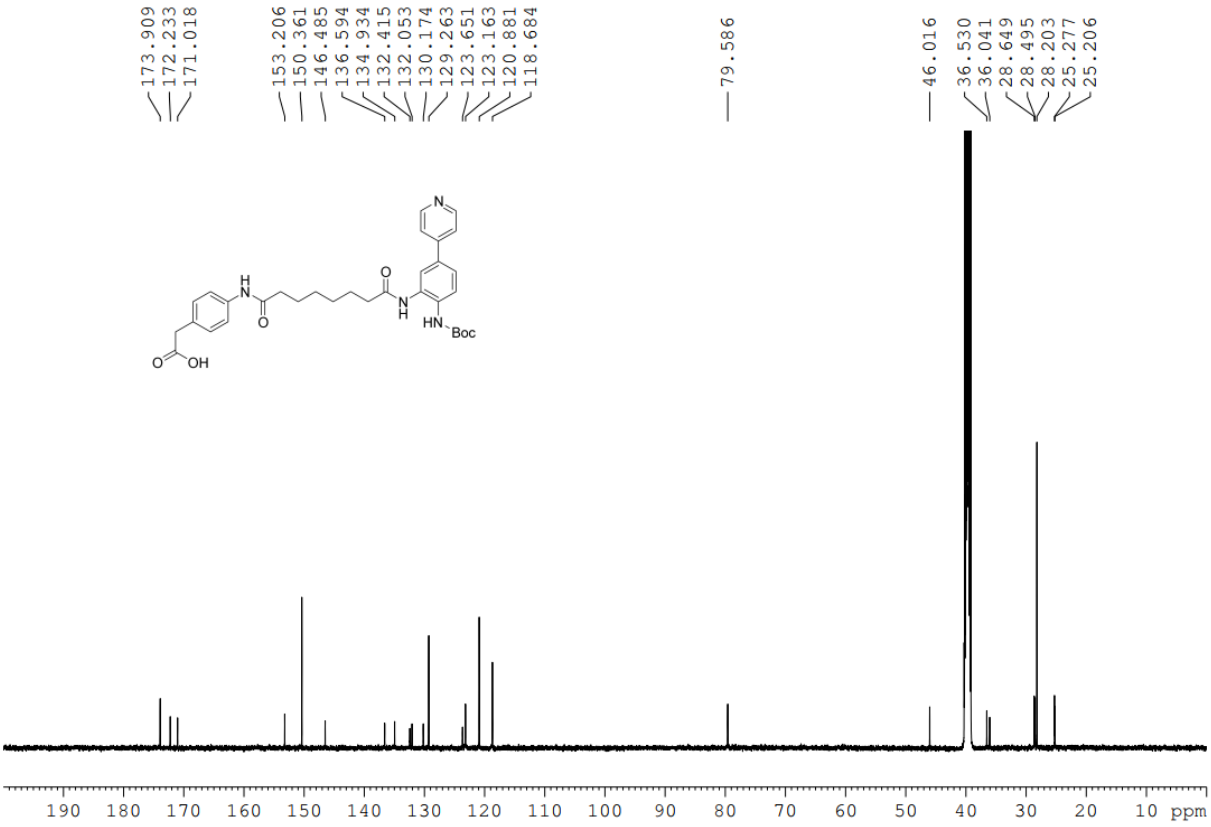

^1H NMR spectrum of **9a** (600 MHz, DMSO-d_6)

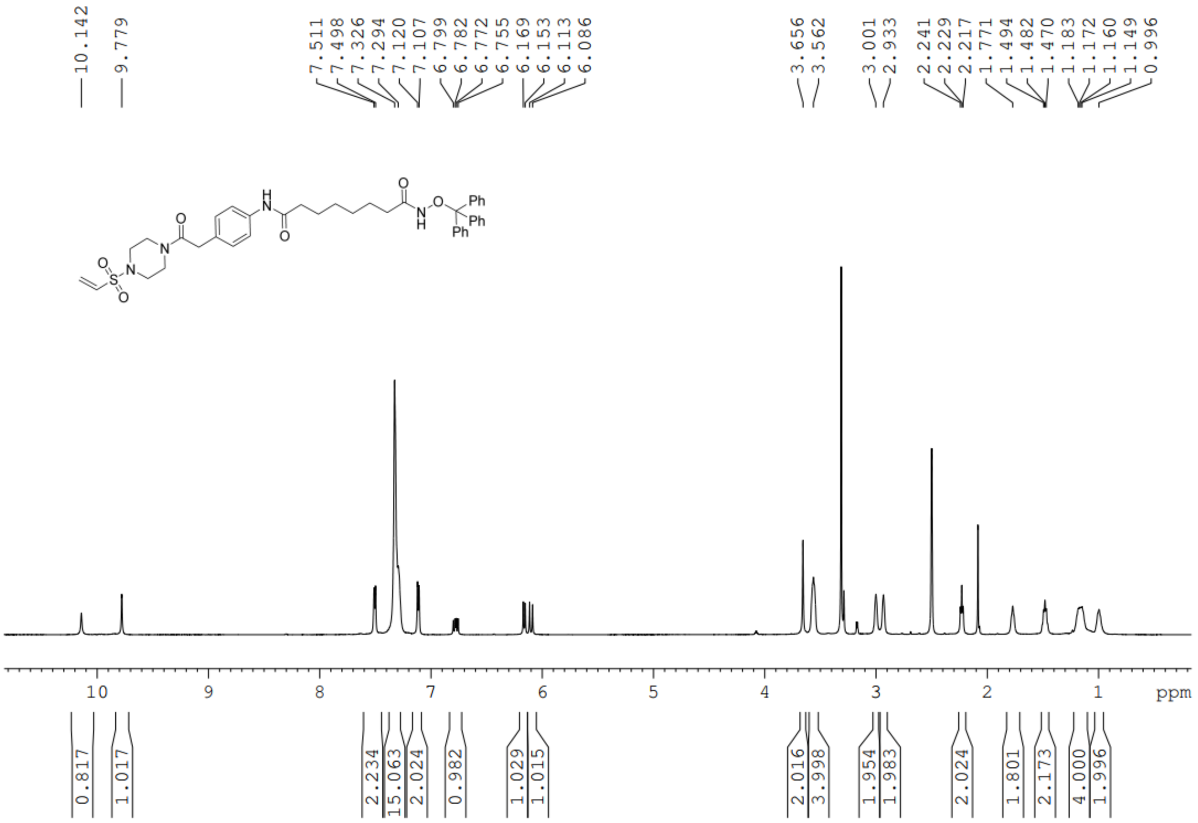

^{13}C NMR spectrum of **9a** (151 MHz, DMSO-d_6)

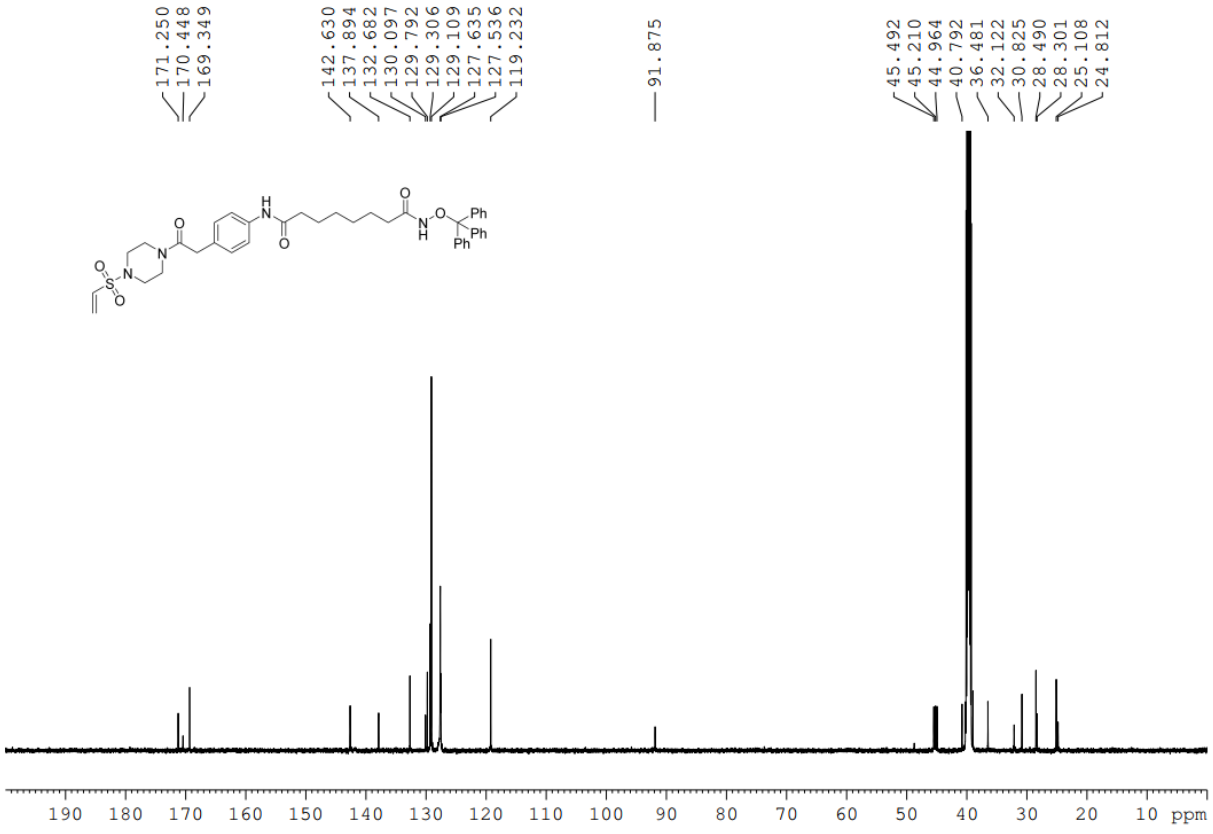

^1H NMR spectrum of **9b** (600 MHz, DMSO-d_6)

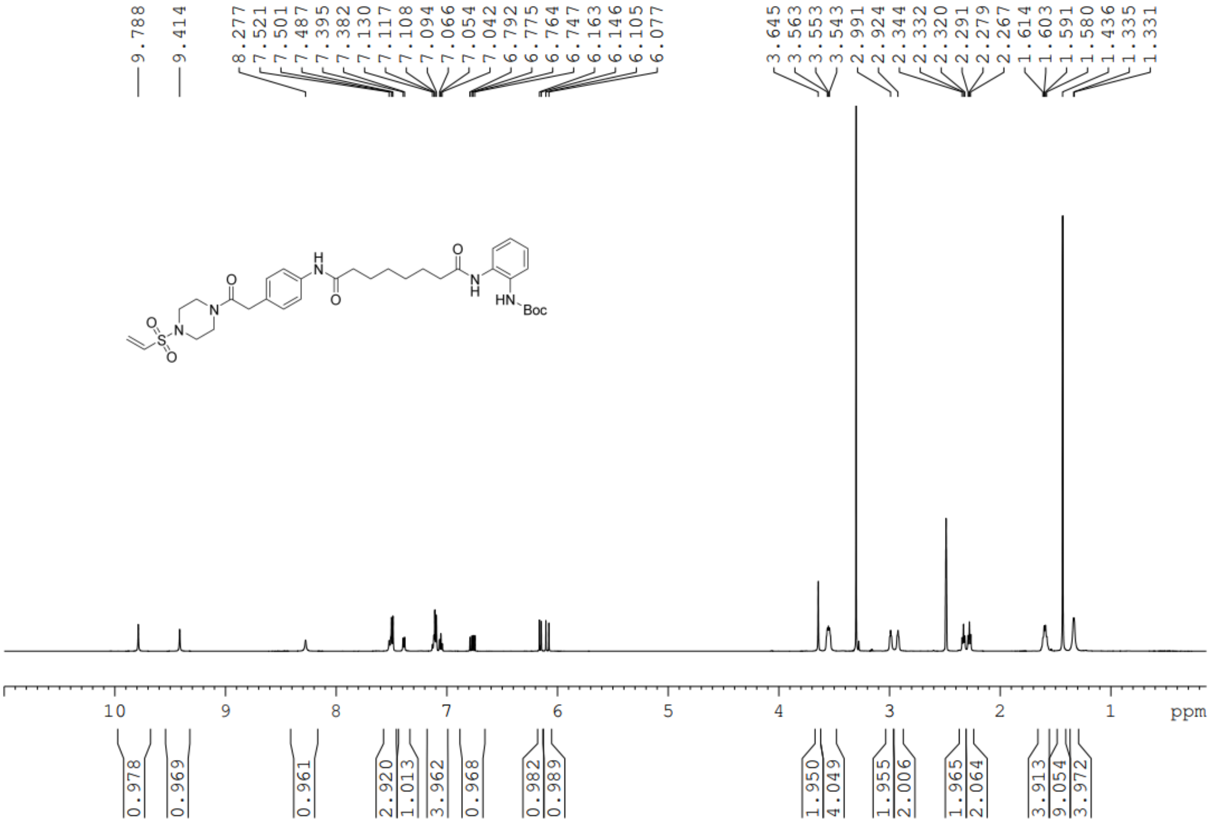

^{13}C NMR spectrum of **9b** (151 MHz, DMSO-d_6)

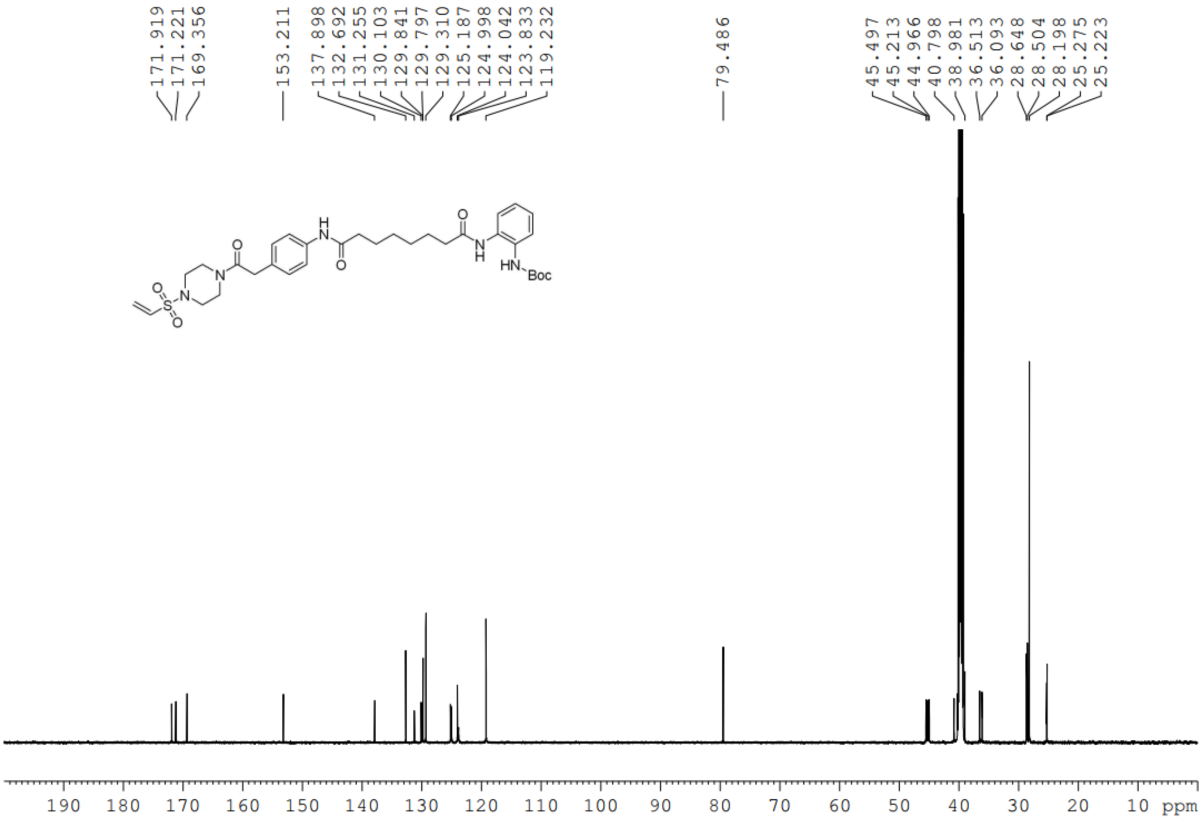

^{1}H NMR spectrum of **9c** (600 MHz, DMSO-d_6)

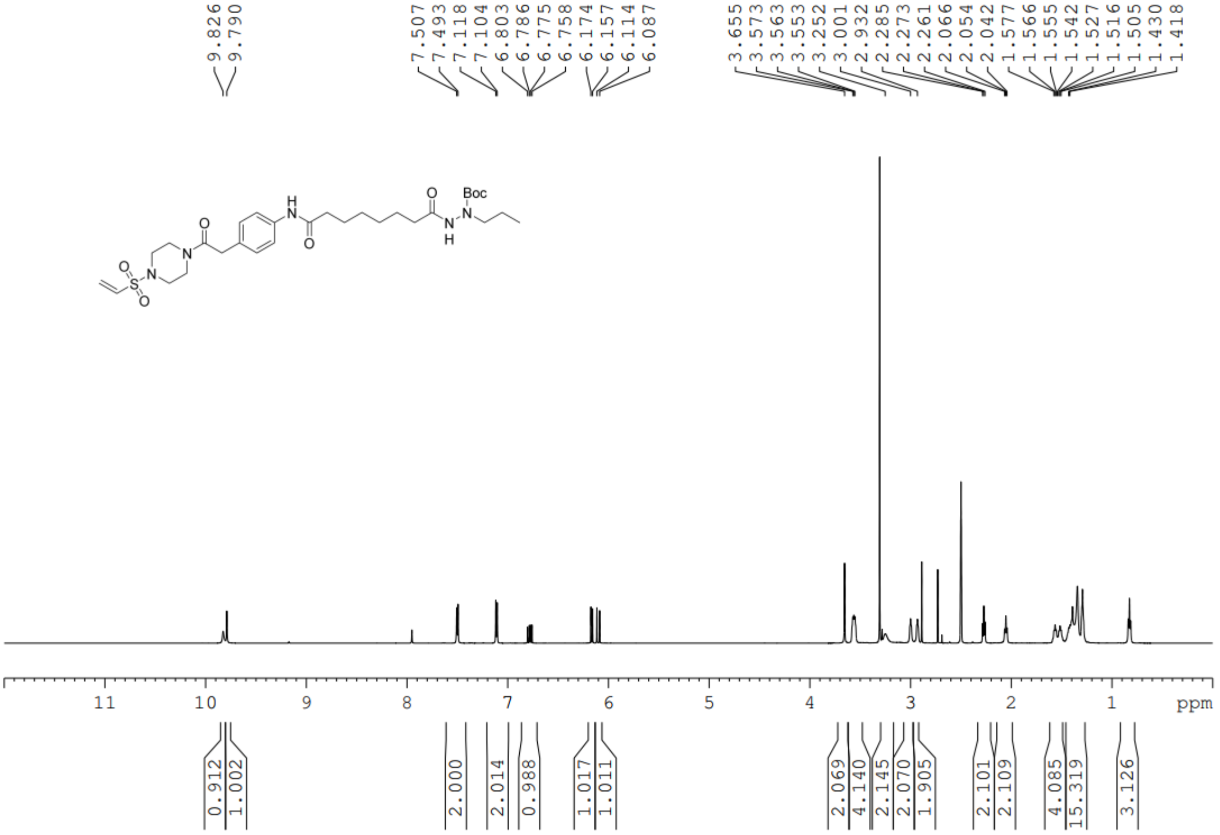

^{13}C NMR spectrum of **9c** (151 MHz, DMSO-d_6)

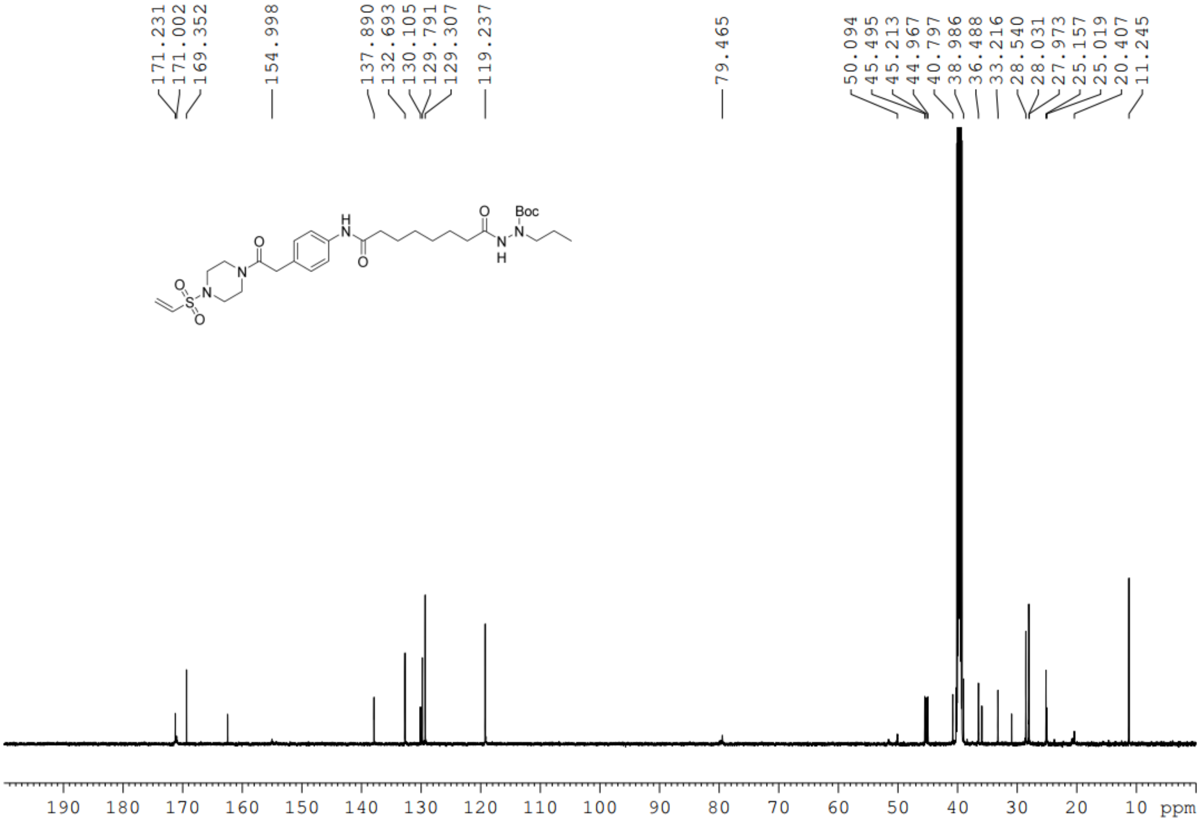

^{1}H NMR spectrum of **9d** (500 MHz, DMSO-d_6)

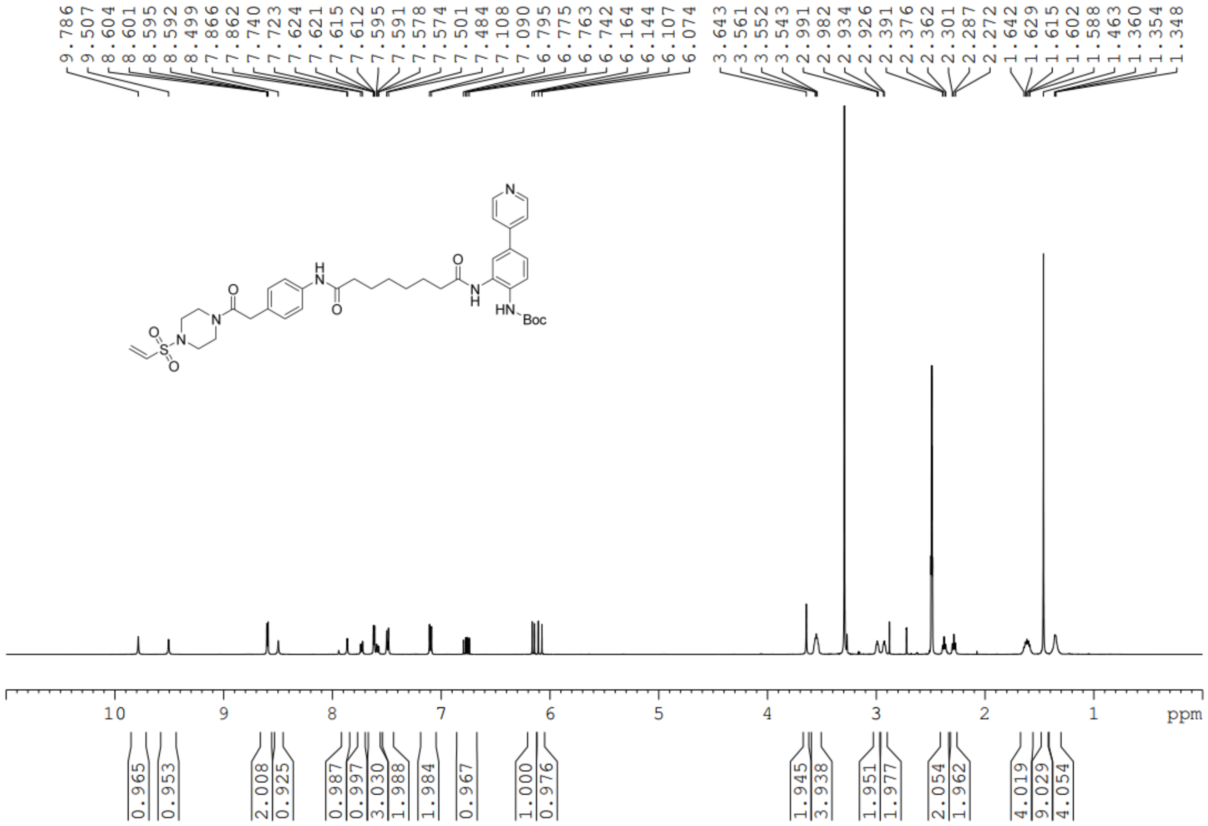

^{13}C NMR spectrum of **9d** (126 MHz, DMSO-d_6)

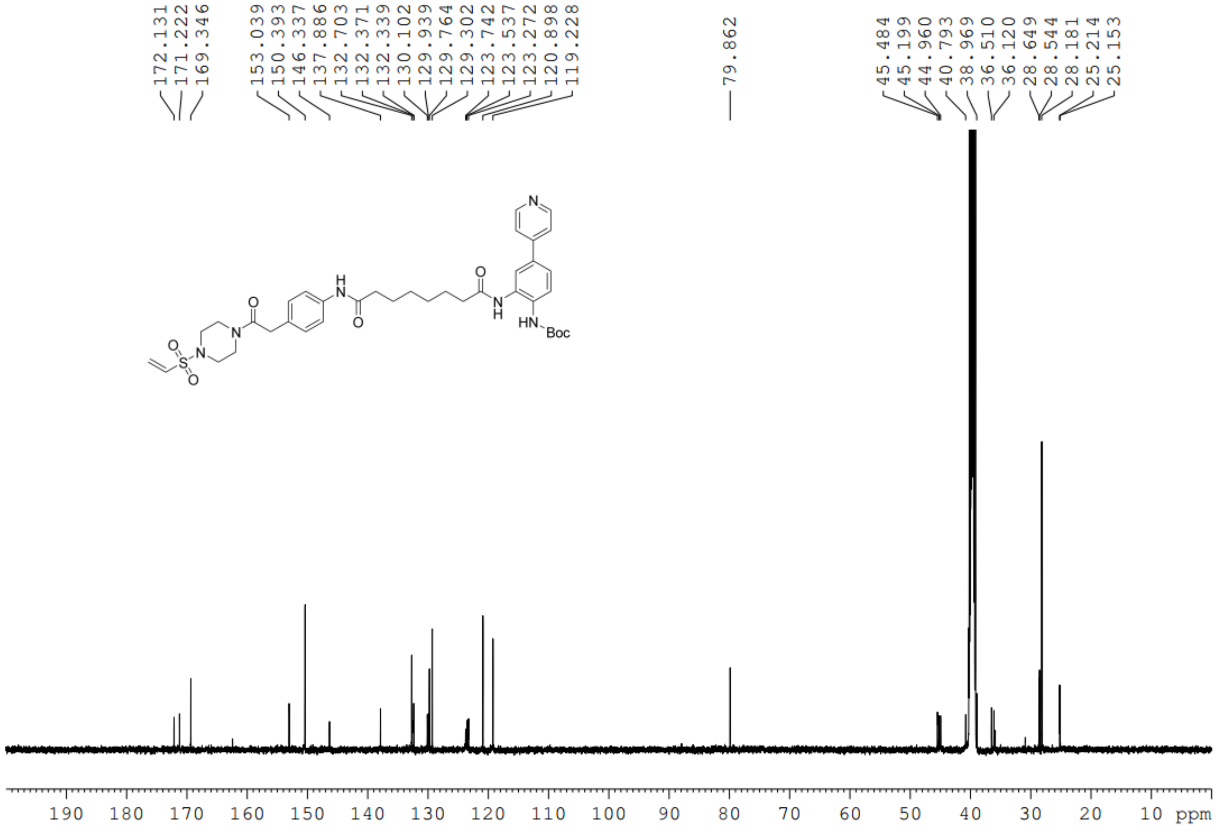

^{1}H NMR spectrum of **10a** (600 MHz, DMSO-d_6)

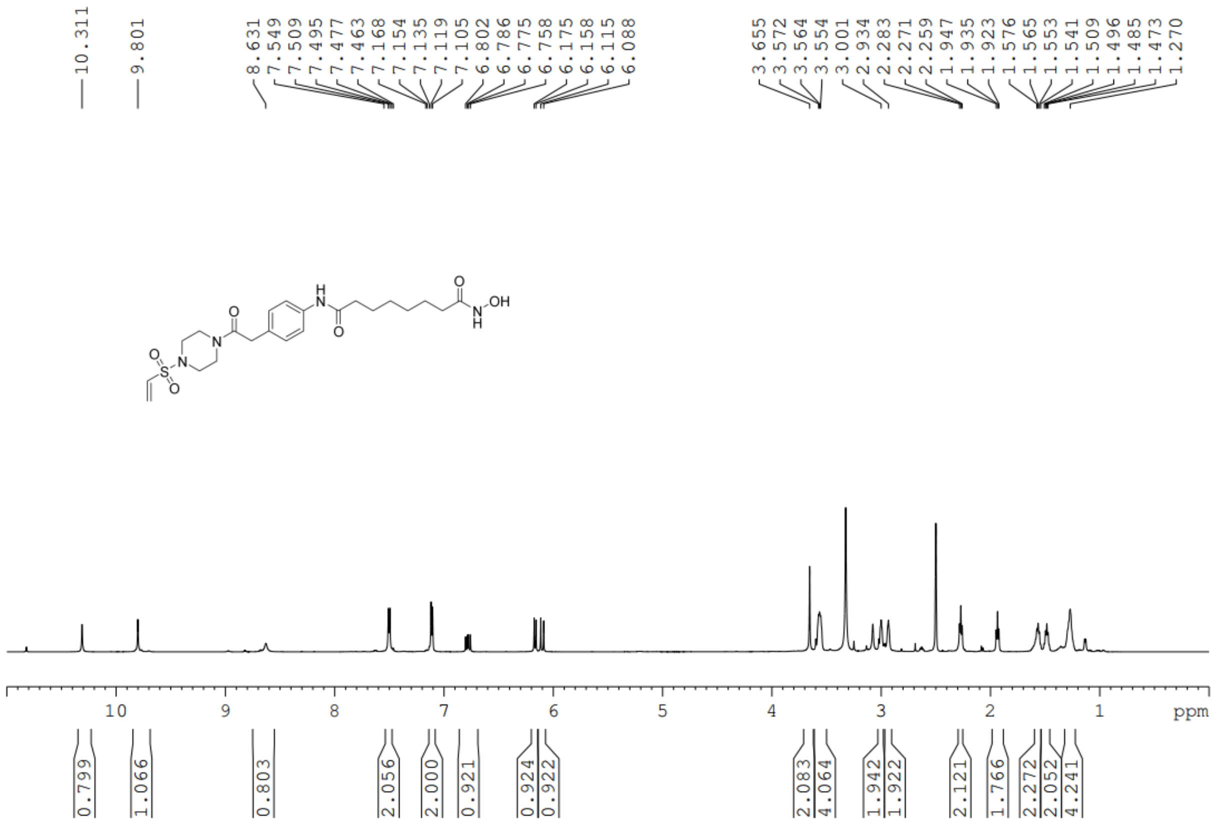

^{13}C NMR spectrum of **10a** (151 MHz, DMSO-d_6)

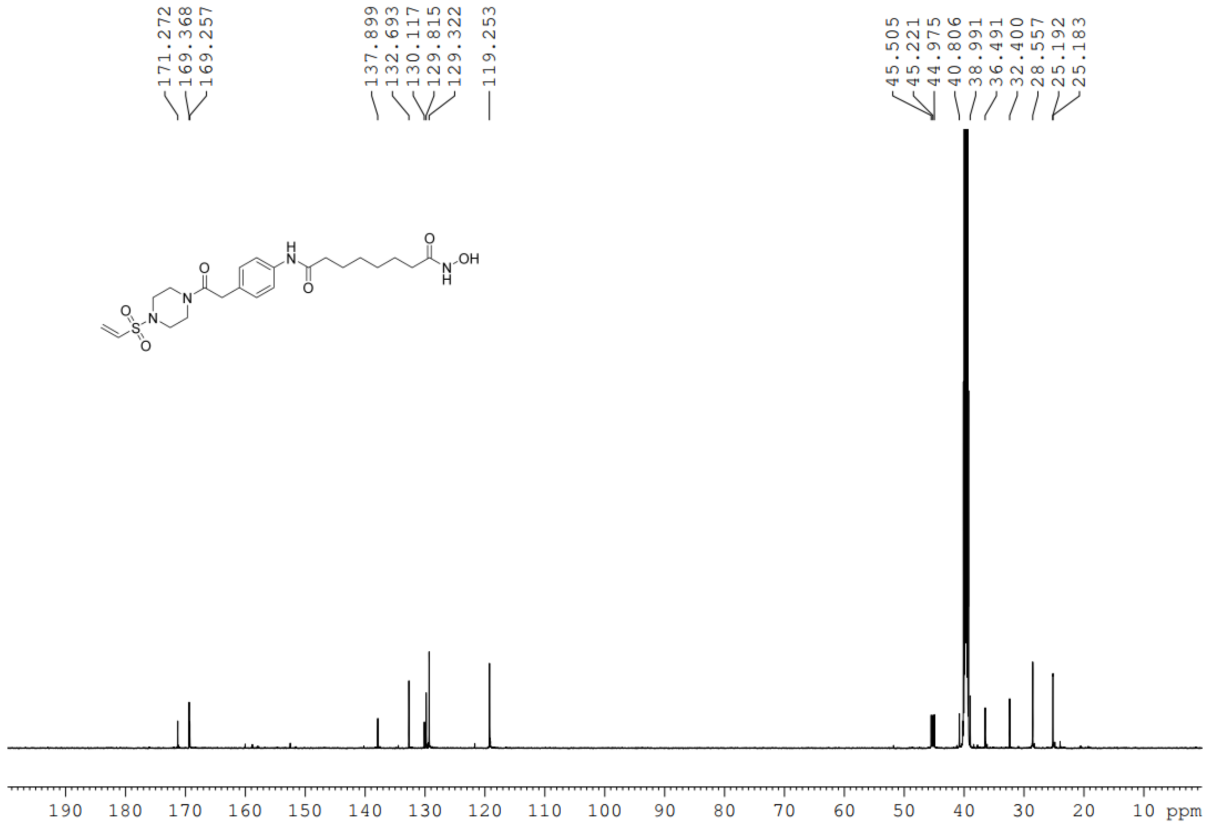

^{1}H NMR spectrum of **10b** (500 MHz, DMSO-*d*6)

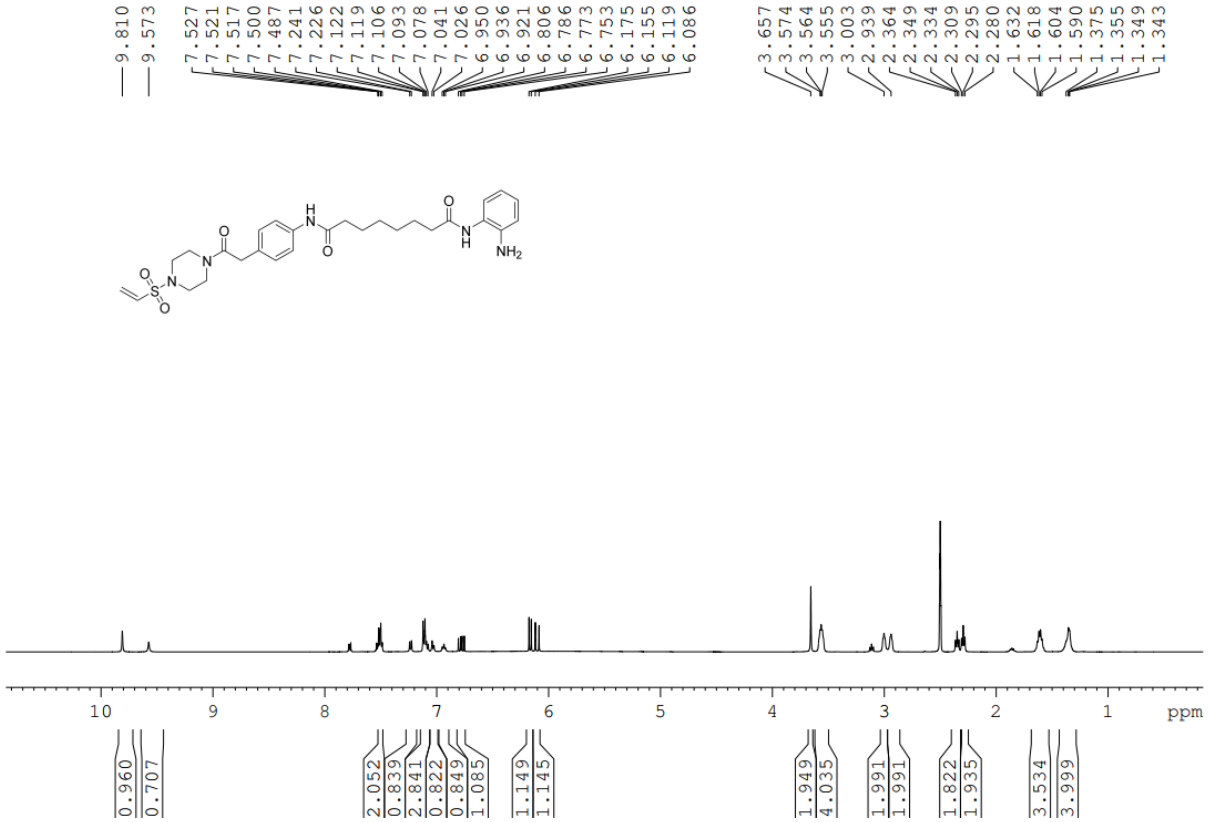

^{13}C NMR spectrum of **10b** (126 MHz, DMSO-*d*6)

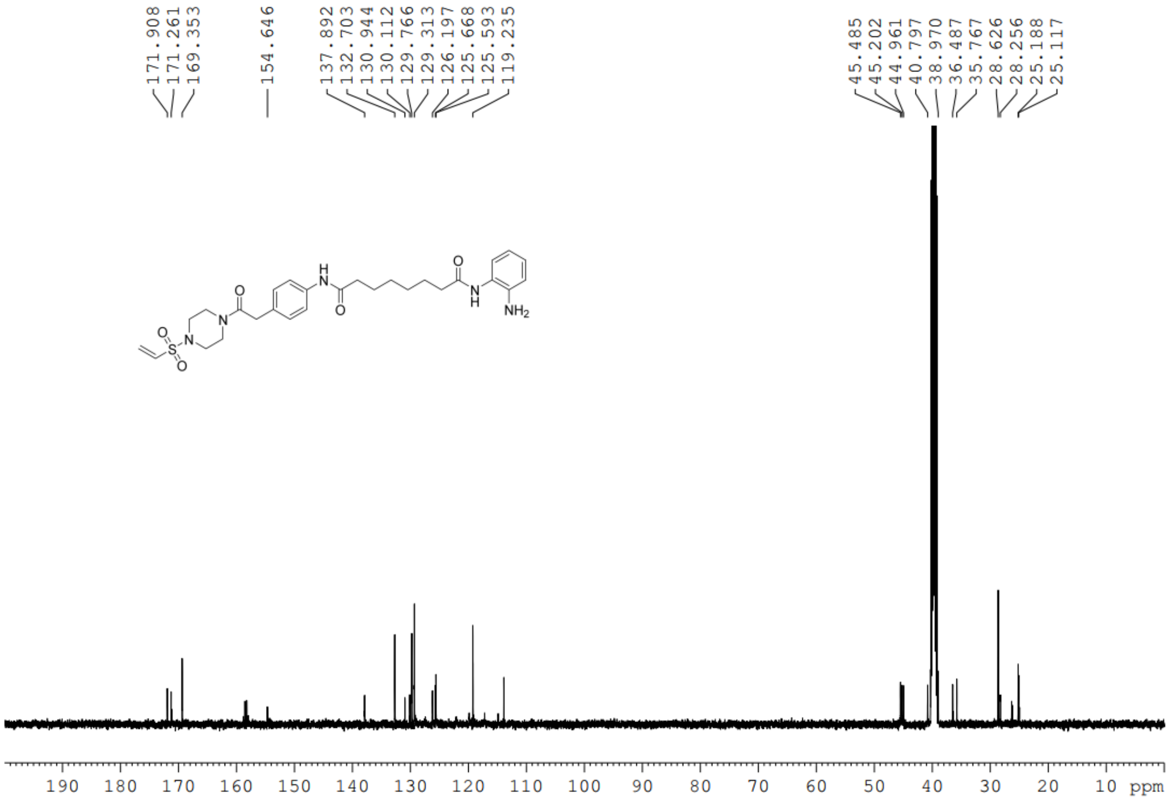

^{1}H NMR spectrum of **10c** (600 MHz, DMSO-d_6)

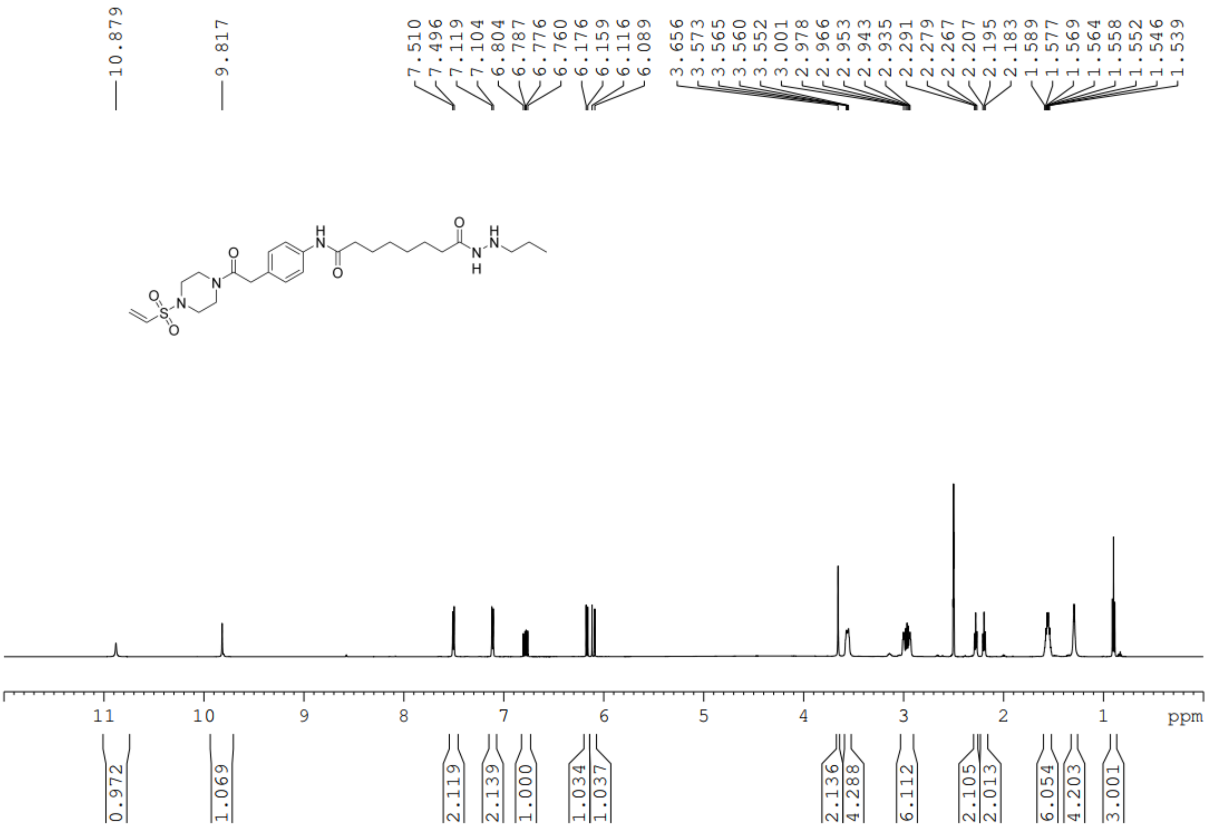

^{13}C NMR spectrum of **10c** (151 MHz, DMSO-d_6)

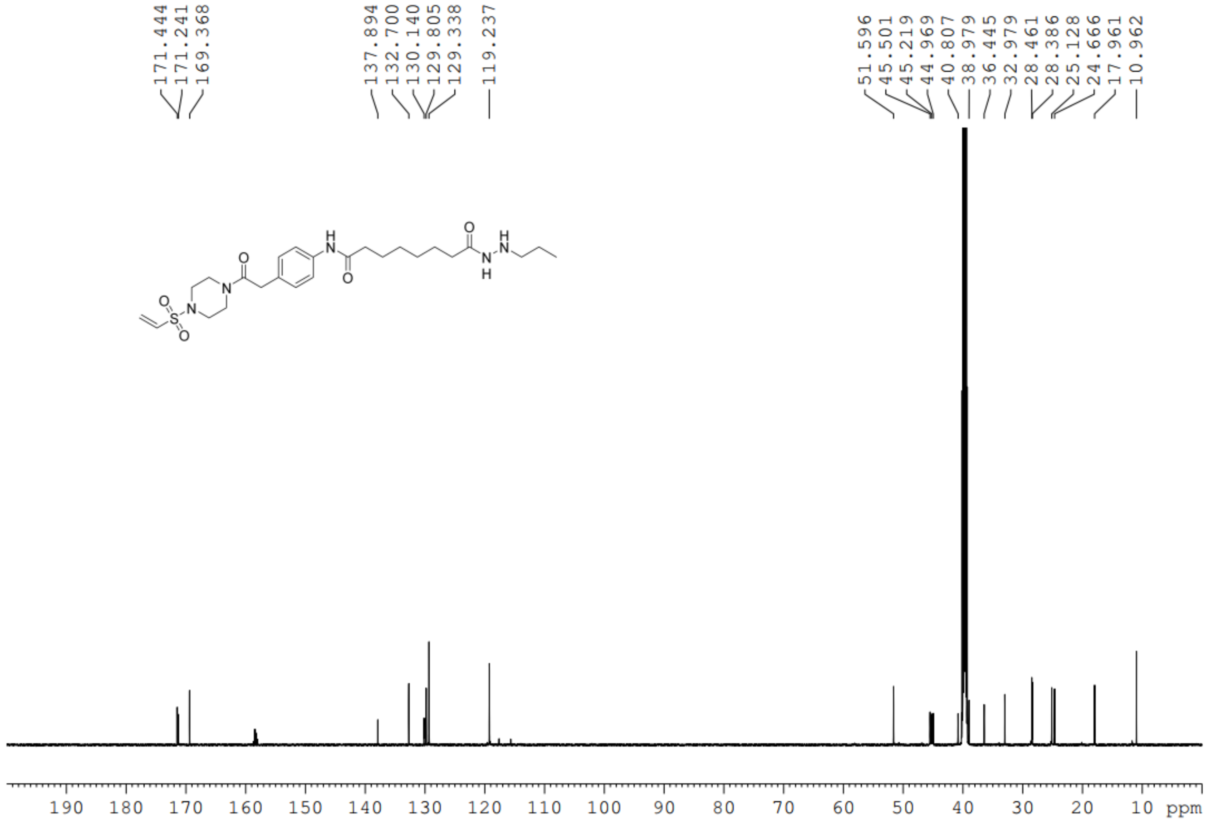

^{1}H NMR spectrum of **10d** (600 MHz, DMSO-d_6)

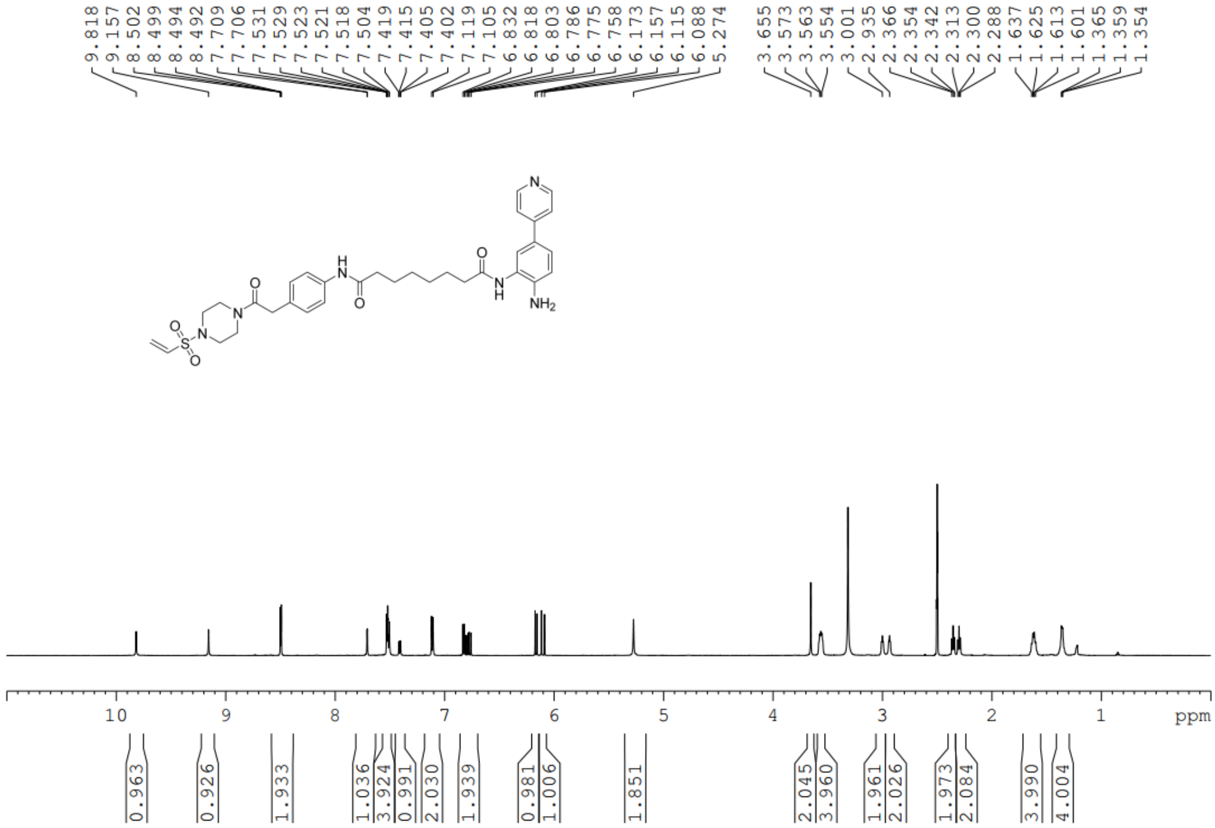

^{13}C NMR spectrum of **10d** (151 MHz, DMSO-d_6)

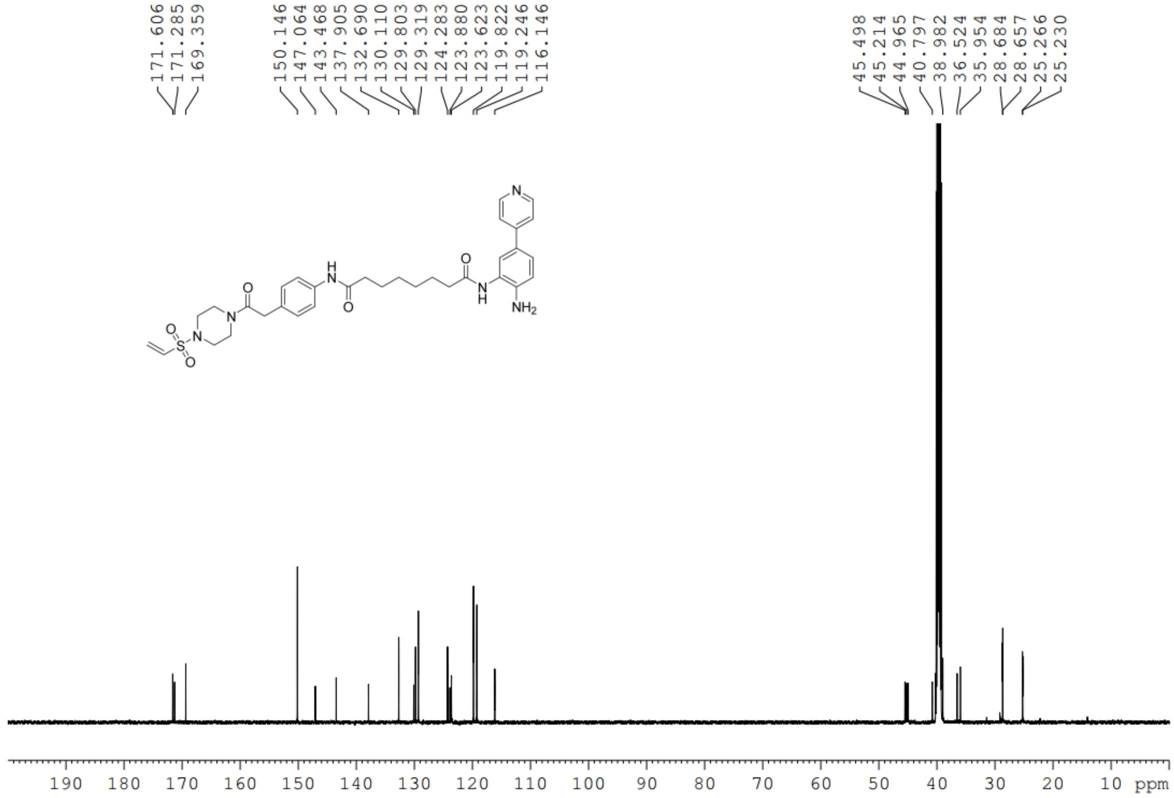

^{1}H NMR spectrum of **10a-nc** (500 MHz, DMSO-d_6)

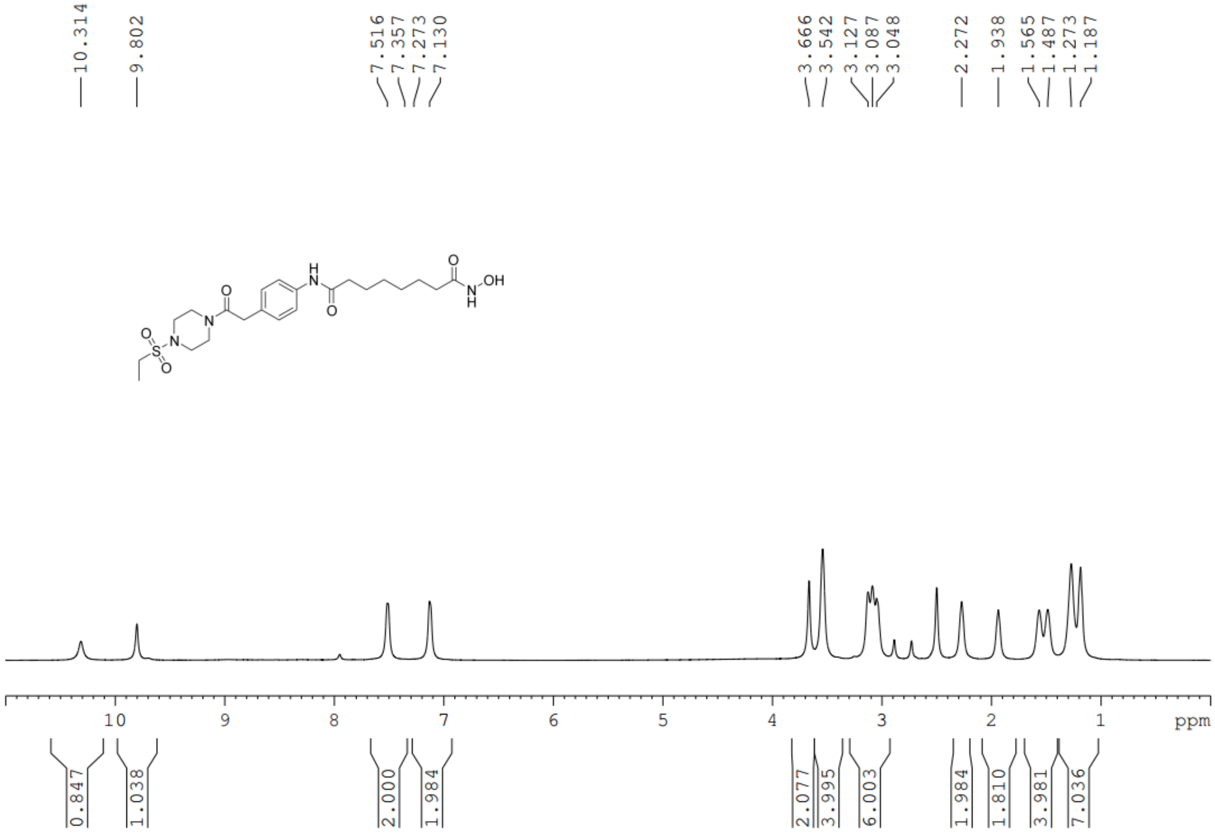

^{13}C NMR spectrum of **10a-nc** (126 MHz, DMSO-d_6)

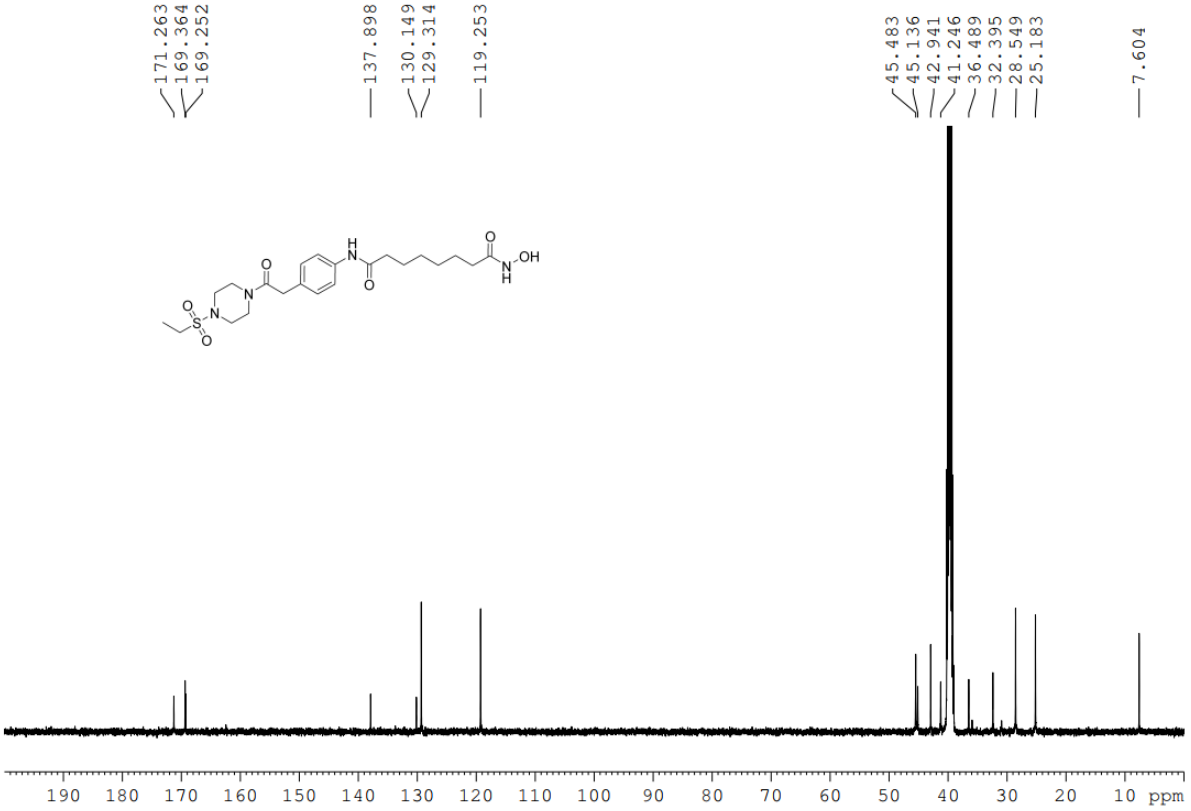

3. HPLC chromatograms

HPLC Chromatogram of **10a**, purity 98.1%.

Chromatogram

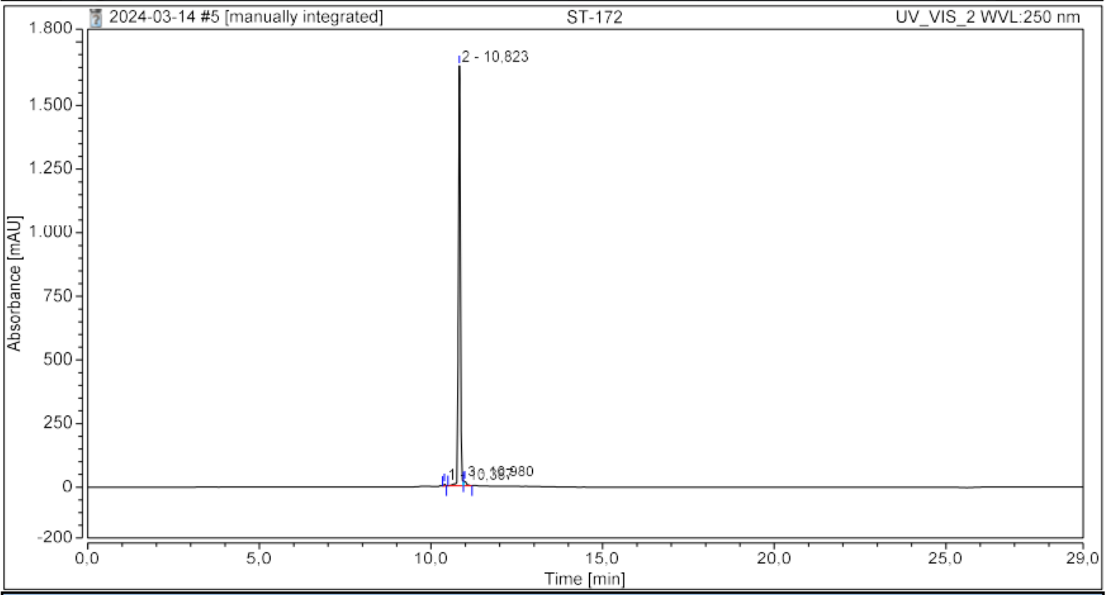

Integration Results

No.	Peak Name	Retention Time<br>min	Area<br>mAU*min	Height<br>mAU	Relative Area<br>%	Relative Height<br>%	Amount<br>n.a.
1		10,387	0,422	9,109	0,39	0,54	n.a.
2		10,823	105,390	1650,672	98,09	98,37	n.a.
3		10,980	1,627	18,170	1,51	1,08	n.a.
**Total:**			**107,439**	**1677,951**	**100,00**	**100,00**	

HPLC Chromatogram of **10b**, purity 99.1%.

Chromatogram

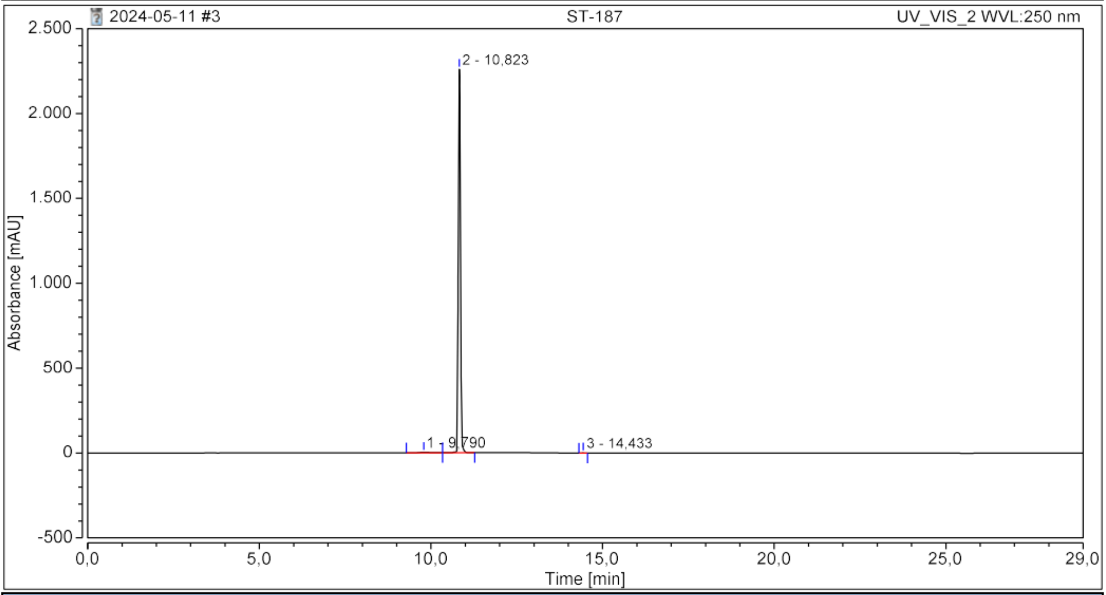

Integration Results

No.	Peak Name	Retention Time<br>min	Area<br>mAU*min	Height<br>mAU	Relative Area<br>%	Relative Height<br>%	Amount<br>n.a.
1		9,790	1,484	3,225	0,91	0,14	n.a.
2		10,823	160,862	2258,957	99,05	99,82	n.a.
3		14,433	0,062	0,895	0,04	0,04	n.a.
**Total:**			**162,408**	**2263,077**	**100,00**	**100,00**	

HPLC Chromatogram of **10c**, purity 97.5%.

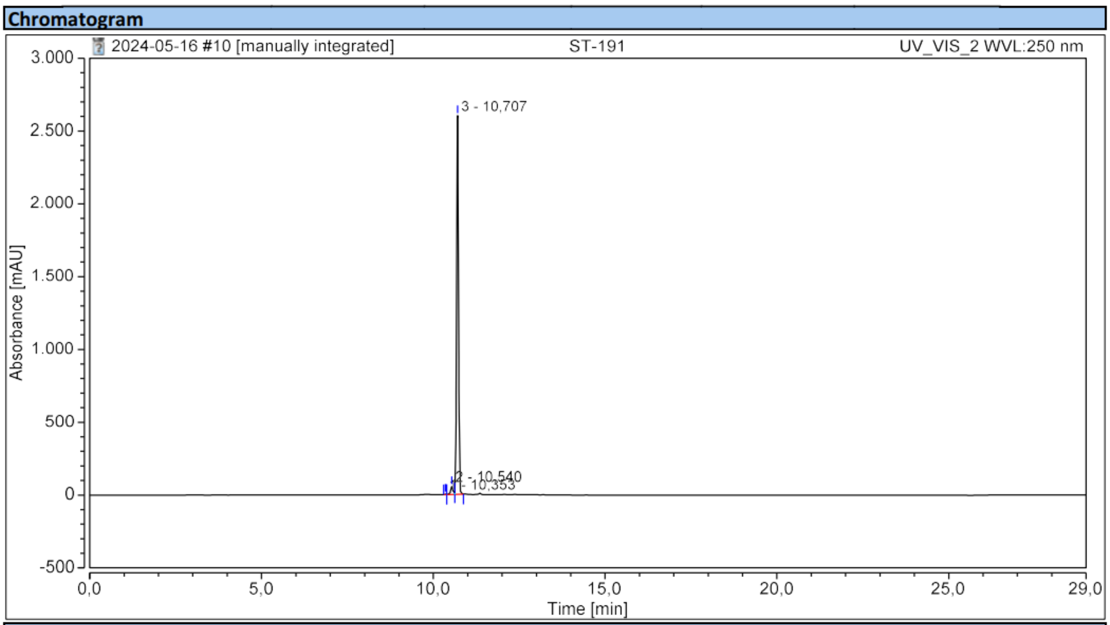

Integration Results

No.	Peak Name	Retention Time min	Area mAU*min	Height mAU	Relative Area %	Relative Height %	Amount n.a.
1		10,353	0,160	3,049	0,09	0,11	n.a.
2		10,540	4,417	54,727	2,42	2,06	n.a.
3		10,707	177,724	2600,383	97,49	97,83	n.a.
**Total:**			**182,302**	**2658,159**	**100,00**	**100,00**	

HPLC Chromatogram of **10d**, purity 98.3%.

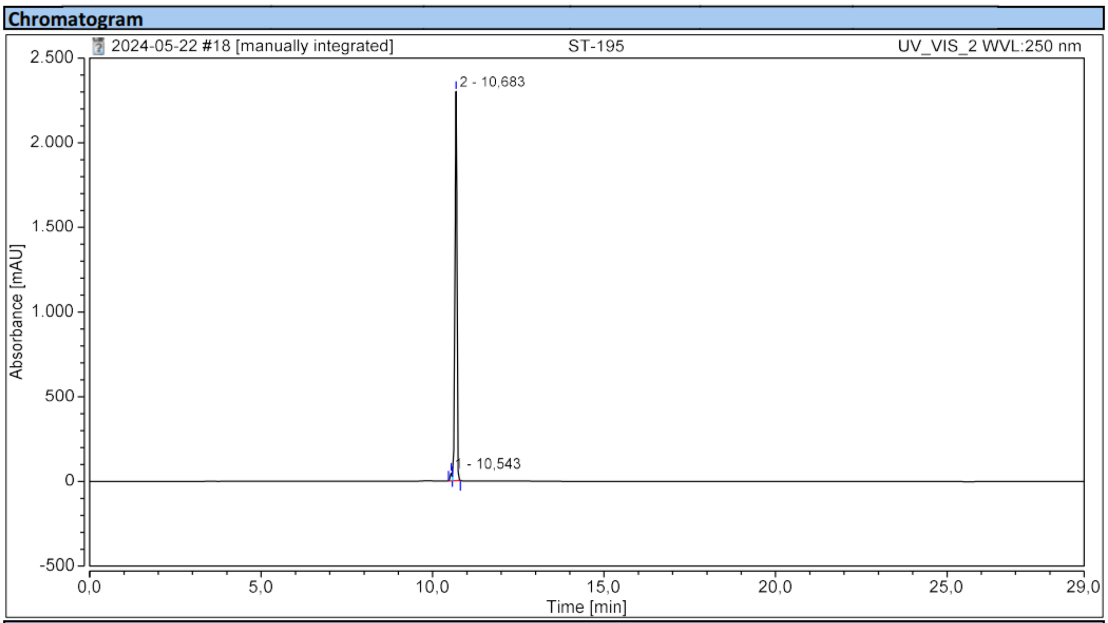

Integration Results

No.	Peak Name	Retention Time min	Area mAU*min	Height mAU	Relative Area %	Relative Height %	Amount n.a.
1		10,543	2,555	45,033	1,70	1,92	n.a.
2		10,683	147,677	2298,952	98,30	98,08	n.a.
**Total:**			**150,232**	**2343,985**	**100,00**	**100,00**	

HPLC Chromatogram of **10a-nc**, purity 98.3%.

Chromatogram

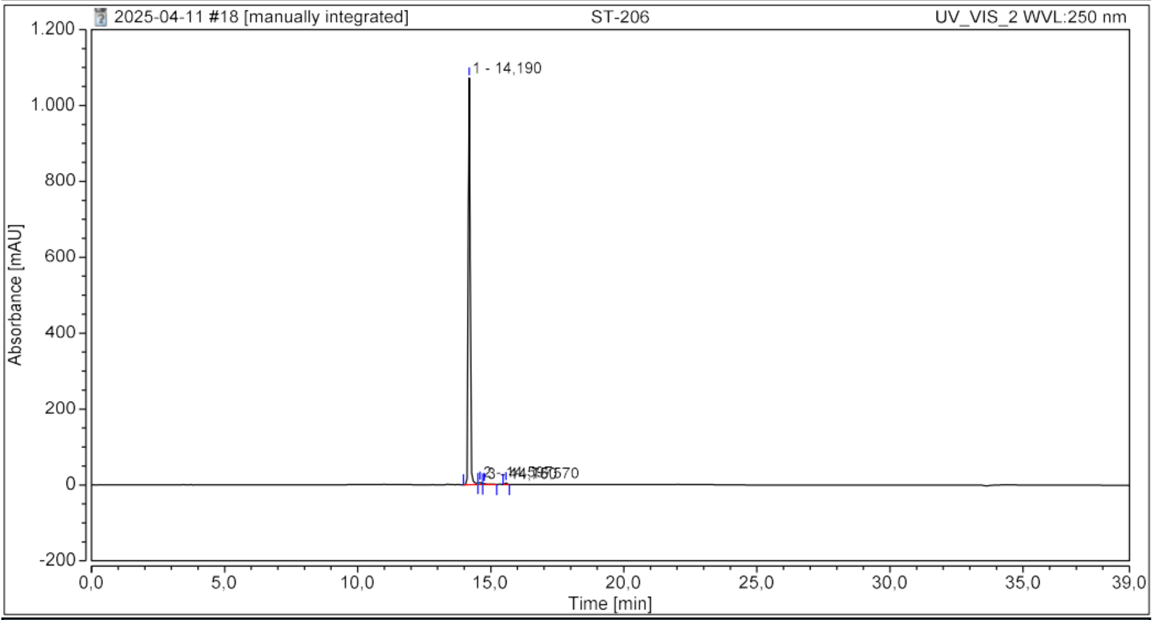

Integration Results

No.	Peak Name	Retention Time min	Area mAU*min	Height mAU	Relative Area %	Relative Height %	Amount n.a.
1		14,190	85,106	1071,778	98,26	98,72	n.a.
2		14,597	0,708	6,528	0,82	0,60	n.a.
3		14,760	0,418	2,690	0,48	0,25	n.a.
4		15,570	0,379	4,727	0,44	0,44	n.a.
**Total:**			**86,611**	**1085,723**	**100,00**	**100,00**	
